# Integrated in vitro and in vivo evaluation of ivermectin hydrogel formulation for management of scabies with pharmacological assessment

**DOI:** 10.1038/s41598-026-53626-w

**Published:** 2026-06-04

**Authors:** Sara Abdel-Aal Mohamed, Alzahraa Abdelraouf Ahmad, Fatma El-Zahraa A. Mustafa, Eman A. Negm, Dalia Hassan, Ahmed Mohammed, Ahmed K. Hassan, Mahmoud S. Sabra, Jelan A. Abdel -Aleem, Ahmed Abdelfattah, Marwa A. Sayed

**Affiliations:** 1 Department of Parasitology, Faculty of Veterinary Medicine, Assiut University, Assiut, 71526 Egypt; 2 Department of Parasitology, Faculty of Medicine, Assiut University, Assiut, 71515 Egypt; 3 Department of Cell and Tissues, Faculty of Veterinary Medicine, Assiut University, Assiut, 71526 Egypt; 4 Department of Physiology, Faculty of Veterinary Medicine, Assiut University, Assiut, 71526 Egypt; 5 Department of Animal, Poultry hygiene and Environmental Sanitation, Faculty of Veterinary Medicine, Assiut University, Assiut, 71526 Egypt; 6 Department of Behavior and Management of Animals, Poultry and Aquatics, Faculty of Veterinary Medicine, Assiut University, Assiut, 71526 Egypt; 7 Department of Animal Husbandry and Livestock Development, School of Veterinary Medicine, Badr University in Assiut, Assiut, Egypt; 8 Department of Avian and Rabbit Medicine, Faculty of Veterinary Medicine, Assiut University, Assiut, 71526 Egypt; 9 Department of Pharmacology, Faculty of Veterinary Medicine, Assiut University, Assiut, 71526 Egypt; 10 Department of Industrial Pharmacy, Faculty of Pharmacy, Assiut University, Assiut, 71526 Egypt; 11 Faculty of Pharmacy and Pharmaceutical Sciences, University of Alberta, Edmonton, AB T6G 2E1 Canada

**Keywords:** *Sarcoptes scabiei*, Ivermectin hydrogel, Acaricidal activity, Oxidative stress, Inflammation, Immunomodulation, Biological techniques, Diseases, Drug discovery, Immunology, Medical research, Microbiology

## Abstract

Scabies is a contagious skin disease caused by *Sarcoptes scabiei *mites, posing a significant health risk, especially in poor, crowded settings. Existing treatments are facing challenges due to poor compliance and side effects, necessitating alternative therapies. Hydrogel formulations offer promise by enhancing drug release and bioavailability. This study tested a novel topical ivermectin (IVR) hydrogel for scabies, evaluating its effectiveness, anti-inflammatory, and antioxidant properties. It also assessed treatment outcomes of skin structure and its effect on animal behavior and locomotor activity. IVR hydrogel (1% w/v) was formulated and evaluated based on its physicochemical properties, *in vitro* release kinetics, and acaricidal activity using a contact bioassay and scanning electron microscopy (SEM). An* in vivo* bioassay was performed using experimentally infested rabbits treated topically with IVR hydrogel. Treatment efficacy was evaluated via histopathology, oxidative stress, inflammatory and immunological markers, and behavioral changes measured through open-field testing. The IVR gel, at a 100% concentration, achieved complete mite mortality *in vitro* within 10 minutes, accompanied by extensive cuticular damage. *In vivo*, treated rabbits exhibited marked clinical improvement, attaining a clinical score near zero (0.17) by day 21, alongside enhanced skin condition and reduced inflammation. The treatment resulted in a statistically significant reduction in inflammatory markers (TNF-α, IL-6, *P<*0.001) and oxidative stress (MDA, *P<*0.001) without significant alterations in specific immunoglobulin levels (IgG and IgE). Histopathological analysis confirmed improvement of skin architecture, while behavioral assessments revealed no adverse effects on animal behavior. IVR hydrogel demonstrated significant, concentration-dependent acaricidal activity, biocompatibility, and effectiveness in reducing oxidative and inflammatory damage. Based on these findings, topical ivermectin hydrogel may offer a promising platform for further investigation as a treatment modality for scabies.

**Supplementary Information:**

The online version contains supplementary material available at 10.1038/s41598-026-53626-w.

## Introduction

Scabies is a highly infectious skin disease caused by the itch mite *Sarcoptes scabiei (S. scabiei)*, which poses a significant global health threat. Scabies is easily transmitted through direct or indirect contact with fomites, with outbreaks frequently reported in overcrowded environments such as jails, boarding schools, and hostels. *Sarcoptes* mites affect various mammals, including humans, domestic animals, and wildlife^1,2^. The prevalence of scabies varies widely from 0.2% to 71.4% in endemic regions, affecting susceptible populations, such as children, the elderly, and immunocompromised persons^3,4^. According to the World Health Organization, it is estimated that over 200 million individuals are affected at any given time worldwide, scabies is considered a neglected disease due to its substantial impact on resource-limited communities, where poverty and inadequate healthcare access exacerbate its spread^4–6^.

Scabies can also cause a slew of secondary infections if left untreated, including significant pyoderma, rheumatic heart disease, and even acute renal infection^7^. Additionally, the incidence of secondary bacterial infections increases due to scratching and skin fissuring^8,9^.

While several topical and oral treatments are available for scabies, their clinical utility is constrained by some factors, including variable efficacy, safety, and incomplete FDA approvals for certain formulations^10^. The FDA has approved 5% permethrin cream as the primary treatment for scabies in individuals aged > 2 months, demonstrating a significantly higher cure rate compared to alternative therapies such as sulfur, lindane, crotamiton, and benzyl benzoate^11^. Despite the effectiveness of permethrin in the treatment of scabies, treatment failures can occur due to improper application, mite resistance to permethrin, reinfestation from untreated contacts or environments, and noncompliance, particularly in cases involving toddlers or hyperkeratotic skin^6,12–14^. These limitations highlight the challenges in achieving consistent therapeutic success with topical agents alone.

Oral ivermectin (IVR), though widely used, is the only systemic option and is utilized with specific cases such as severe crusted or Norwegian scabies or in case of treatment failure to topical medications, or during outbreak control^15–17^. While it offers rapid action and a favorable safety profile, it lacks FDA approval for scabies in many countries, underscoring the need for expanded therapeutic choices^6,18–21^. IVR exerts its antiparasitic effects by modulating glutamate-gated chloride channels in arthropods, leading to paralysis and death^22^. Beyond its antiparasitic properties, IVR demonstrates anti-inflammatory properties, suppressing pro-inflammatory cytokines (IL-1β, IL-6, TNF-α) while enhancing IL-10, thereby alleviating itching and secondary infections^21,23^. However, emerging resistance and contraindications in certain populations (e.g., young children, pregnant women) highlight the need for novel therapeutic alternatives^18^.

Oxidative stress is also an important factor in the development of scabies. Different indicators related to oxidative stress, including lipid peroxidation, free glutathione, and malondialdehyde, exhibit notable variations in disease severity^24^. Furthermore, antioxidants such as catalase, zinc, and copper are decreased in patients with scabies^25^. While systemic IVM is effective in achieving clinical and parasitological cures, some studies report that some histopathological and biochemical changes may persist after treatment^26,27^. Furthermore, IVM treatment was reported to be associated with oxidative stress, leading to persistence of tissue damage and inflammation up to three months after treatment, as reported in some studies^28^. Therefore, improving these properties in IVR formulation may be advantageous in treatment, especially in severe forms of the disease.

While topical permethrin and oral ivermectin remain first-line treatments for scabies, the emerging concerns about drug resistance and the need for better patient compliance are driving a significant revolution in therapy and diagnostics^29,30^. This includes advanced non-invasive diagnostic tools, for instance, ultraviolet dermoscopy, novel therapeutic agents such as moxidectin, and advanced drug delivery systems. In particular, pharmaceutical research is intensely focused on improving topical ivermectin formulations—such as thixotropic gels, sprayable gels, and nano-carrier systems like transethosomes—to enhance skin penetration, reduce systemic side effects, and improve treatment administration, thereby addressing key limitations of conventional therapies^31,32^.

Hydrogel formulations have proven effective in dermatological applications, such as wound healing and anti-inflammatory therapy^33,34^. They enhance drug penetration through the skin and improve patient compliance. Due to their biocompatibility, controlled drug release, and ability to retain moisture, hydrogel formulations are promising for delivering active agents to the skin^35^. While oral IVR remains systemically superior, topical formulations may have fewer side effects related to persistent pruritus^36^. However, their use in severe infections or mass treatment is limited due to application challenges such as the need for uniform coverage over large skin areas, patient compliance, especially in overcrowded or resource-limited settings, and difficulties in dosing and application during outbreaks^37^. Further research is needed to enhance hydrogel delivery for broader clinical use.

Therefore, developing innovative treatment strategies for scabies should be a priority to effectively tackle the ongoing challenges related to its prevention and management. This study evaluated the efficacy of a topical IVR hydrogel formulation against *S. scabiei* mites through in vitro and in vivo assays, with a focus on its anti-inflammatory and antioxidant properties. Additionally, we assessed its impact on skin structure restoration and behavioral outcomes in treated animals. This innovative formulation could serve as a promising alternative or complementary option for managing scabies, especially in cases where systemic IVR is impractical.

## Materials and methods

### Ethics guidelines for the protocol

The study protocol adhered to ethical and animal care guidelines established in accordance with the ARRIVE guidelines^38^. The ethics committee of the Faculty of Veterinary Medicine, Assiut University, approved the experiment (approval number 06/2023/0101).

### Chemicals

Ivermectin pure powder was purchased from Amoun Pharmaceutical Company (Cairo, Egypt). Hydroxypropylmethyl cellulose (HPMC) was purchased from Aldrich Chem. Co., USA. Sodium alginate (Na-alginate) was purchased from Judex Laboratories Reagent, UK, and Carbopol 934 was purchased from C.P. Evan Co., England. All other chemicals were of analytical grade. Standard cellophane membranes (molecular weight cut-off range equals 12000) were purchased from Sigma Chem. Co., USA. Permethrin 5% was purchased from the International Co. for Pharmaceutical Industries of Arab Perfumes, Chemicals & Pharmaceutical Company (Egypt).

### Collection of mites

*S. scabiei* mites were collected from rabbits with scabies at Assiut University Animal Farm, Egypt. The infested rabbits showed severe signs of sarcoptic mange, as evidenced by microscopic examination of the skin scrapings. Live mites were obtained by scraping the infected rabbit’s crust with a scalpel. The mites were examined microscopically and placed in a sterile Petri dish for the in vitro assay^39^.

### Compatibility studies

#### Fourier transform infrared (FT-IR) studies

FT-IR spectroscopic study was conducted to determine the possible interactions between Ivermectin and the used polymers. Ivermectin alone, sodium alginate, Carbopol 934 and hydroxyl propyl methyl cellulose (HPMC), each alone as well as the physical mixtures of the drug and polymer (1:1 w/w) were subjected to Fourier Transform Infrared Spectroscopy (FT-IR) (FT-IR-470 Fourier Transform Infrared Spectroscopy Shimadzu Corporation, Japan). An amount of (2–3 mg) was mixed with potassium bromide, then compressed into a disc at 4-ton pressure. FT-IR absorption spectra were recorded using FT-IR spectrophotometer Nicolet 6700 over a range of 400–4000 cm-1. Data management was performed using Omnic software^40^.

### Ivermectin gel formulation

The formulation was prepared by dissolving the necessary amount of the drug (1% w/v) in 100 mL of phosphate buffer solution (pH 6.8) using a magnetic stirrer (Human Lab.co, HS-18, Korea). The appropriate amounts of each polymer, as described in Table 1, were gradually added while continuously stirring to ensure the formation of clear gels, while taking care to avoid any clumping of the polymer^41^. Carbopol 934 was mixed with water and homogenized for half an hour using a magnetic stirrer, after which it was left to equilibrate for 24 h. The pH was subsequently adjusted to 5–7 using triethanolamine^42^. Subsequently, we employed sonication for 15 min to efficiently eliminate any trapped air bubbles. The compositions of the formulated gels are listed in Table 1.

**Table 1:** Composition of the prepared ivermectin gel formulation.

Composition (mg)	V1	V2	V3	V4	V5	V6
Ivermectin	0.1	0.1	0.1	0.1	0.1	0.1
HPMC	3	6	---	---	---	---
Carbopol 934	---	---	0.5	1	---	---
Na alginate	---	---	---	---	5	8
Water (ml) to	100	100	100	100	100	100

### Evaluation of the ivermectin hydrogel

#### Organoleptic properties

The hydrogel was visually assessed for color, homogeneity, transparency, and the presence of any lumps.

#### Drug content determination

To evaluate drug content, a precisely weighed 1-g sample of the gel formulation was dissolved in phosphate buffer solution (pH 6.8) using a magnetic stirrer to ensure the drug was completely dissolved. The resulting solution was quantitatively transferred into a 100 mL volumetric flask and further diluted with phosphate buffer. The drug absorbance was measured at a peak wavelength of 245 nm (λ max) using a UV-Visible Spectrophotometer (Jenway-model 6305, England), and the drug content percentage for each gel formulation was determined^41^.

#### PH measurements

The pH of the formulations (V1–V6) was measured using a digital pH meter (Jenway-model 3310, England). The results are presented as the mean of three measurements^41^.

#### Spreadability

For the spreadability assessment, a 0.25-g sample from each gel was placed between two glass slides and allowed to sit undisturbed for 5 min to avoid additional spreading. The upper slide was then secured to a string connected to a fixed weight that was threaded over a pulley. The weight was permitted to hang freely, causing the upper glass slide to slide off. The time taken for the slide to come loose was recorded, with shorter separation times indicating improved spreadability^41^.

#### Rheological studies

The rheological behaviour of the prepared formulas was determined using a Brookfield Viscometer (Brookfield Engineering Laboratories, Inc., USA) with a spindle number of 95 at 25 °C. The spindle was kept rotating for 1 min before the viscosity was measured. The viscosity of the formulas was assessed under various speeds ranging from 10 to 100 rpm. The tests were performed in triplicate, and the mean was calculated^42^.

#### In vitro release of ivermectin gels

The in vitro release of ivermectin from the gels was assessed by dialysis. A 0.5 g sample of each formulation was weighed and placed on a pre-soaked, semipermeable cellophane membrane in phosphate buffer (pH 6.8). The sample covered a circular area of 2.5 cm in diameter with a surface area of 4.9 cm². The membrane, functioning as the donor compartment, was secured over the end of a glass tube (2.5 cm in diameter) with a rubber band to ensure a watertight seal. The tube was immersed in a beaker (receiver compartment) with 100 mL of phosphate buffer (pH 6.8) at 37 ± 0.5 °C in a thermostatic shaker (Dihan Scientific, WSB 45, Korea) at 50 rpm for 2 h. At intervals of 0.25, 0.5, 1, 1.5, and 2 h, 5 mL samples were withdrawn and replaced with fresh buffer to maintain a constant volume. The samples were analyzed spectrophotometrically at λ max 245 nm to determine ivermectin content, with all experiments conducted in triplicate for averaging^43^.

#### Kinetic analysis of the in vitro release data of the drug

To investigate the drug release mechanism from the formulated gels, the in vitro release results were analyzed using several kinetic models, including zero-order, first-order, and Higuchi diffusion models^44^.

### In vitro mortality assay and scanning electron microscopy examination

The in vitro assays began within 2 h of collecting the mites. For each experiment, 20 live active motile mites, consisting of a mix of adults, nymphs, and larvae, were placed in a 3 cm diameter plastic Petri dish^45^. The mites were tested in triplicate experiments with diluted ivermectin gels at various concentrations (100%, 50%, 25%, and 10%). Dilution was carried out in paraffin oil just before the bioassay was established. The negative controls used in the experiment were paraffin oil and a gel base, while the positive control mites were treated with 5% permethrin. 200 µl of each diluted solution, and the controls were used to test the S. scabiei mites. All mites were incubated at 27 ± 2 °C and 75 ± 5% rlative humidity and observed under a stereomicroscope at 5 min, 15 min, 30 min, 60 min, then every hour to 3 h post-treatment. The mites were regularly stimulated with a needle to assess their viability. When they did not respond to stimulation, they were considered dead^46^.

For scanning electron microscopy analysis (SEM), the treated mites were collected and preserved in 2.5% buffered glutaraldehyde solution overnight at 4 °C. After being spun at 1500 rpm for 5 min, the supernatant was removed, and the samples were preserved in 1% osmium tetroxide at 4 °C for 1 h. Then the specimens underwent dehydration through a series of increasing acetone concentrations (30–100%). Afterward, the specimens were dried, placed on stubs, and coated with gold according to the standard procedure^47^. The specimens were photographed at the Unit of Scanning Electron Microscope at Assiut University using a Zeiss DSM 940 electron microscope.

### In vivo bioassay

The present study involved 24 rabbits of the native strain (Egyptian local breed), aged between 2 and 3 months and weighing between 1.5 and 2 kg, and randomly divided into four groups (sex rabbits each) using a random number generator in Microsoft Excel after baseline ectoparasites and helminths screening before the study to ensure uniformity in health status across groups. The first group was designated as the negative control group (non-infested and non-treated). Rabbits in the infected groups were experimentally infested according to the method described by Casais et al., with modifications^48^. The infestation was carried out by direct contact for a 72-h period with a naturally infested rabbit showing severe crusted lesions on the limbs, ears, and nose, confirmed by microscopic examination, simulating the natural process of infestation. Animals were observed for 3 weeks to ensure infestation microscopically and clinically by observing pruritus and skin lesion development. Then, animals were randomly allocated into a positive control group (infected untreated group), an IVR-treated group, and a subcutaneously injected group with commercial IVR 1% (Ivomec, Merck Sharp & Dohme Agvet Inc.) at a dose of 250 µg/kg body weight (bw), administered as two doses within a two-week interval. Additionally, the IVR gel-treated group was treated topically with IVR hydrogel preparation at a neat formulation.

The experiment was conducted at the Parasitology Department of the Faculty of Veterinary Medicine at Assiut University. Throughout the experiment, the groups were housed separately with unrestricted access to water and a standard diet consisting of commercial pellets while maintaining a 12-h light and 12-h dark cycle to help them acclimate to their surroundings. Treatment was applied daily during the first week and every other day for the following week (3 doses/week). The gel formulation was applied with a cotton swab soaked in 2.5 ml of the gel preparation (1% w/v) to form a thin film over the lesions^49^. The animals were followed for 21 days, then euthanized after an intraperitoneal injection of a ketamine-xylazine mixture (90 mg/kg body wt. ketamine and 10 mg/kg body wt. xylazine) at the end of the experiment for further evaluation.

### Clinical evaluation

A clinical assessment was performed to assess whether the sarcoptic skin lesions had deteriorated or healed. The efficacy of the treatments was evaluated by assessing the reduction of skin lesions and the absence of viable mites on microscopic examination at the end of the experiment. Clinical evaluation assessed the severity of *Sarcoptes* lesions in experimentally infested rabbits by parameters used to evaluate the clinical score of infection and degree of recovery based on the clinical score used by Deger and Ural^50^. Table 2 shows the clinical score of infection and the degree of recovery, ranging from 0 to 5. Assessments were performed daily during the first week post-treatment and at the end of the second and third weeks^51^.

**Table 2:** shows the clinical score of infection and the degree of recovery in *Sarcoptes*-infested rabbits.

Infection and degree of recovery	Clinical score
Absence of clinical signs of scabies.	0
Itching without other clinical lesions observed.	1
Erythema without other lesions of scabies.	2
Small number of fresh lesions (erythematous rash, papules and thin scabs).	3
Moderate lesions (papules, hyperkeratosis, and crusts).	4
Severe lesions (extensive fissured crusts, haemorrhage and alopecia).	5

### Serological and biochemical assays

Blood samples were collected from all rabbits at the end of the experiment. Five millilitres of blood were obtained from the rabbit’s ear vein. The samples were then centrifuged at 2500 rpm for 20 min, and the obtained serum was stored at −20 °C for further tests. The inflammatory markers tumor necrosis factor alpha (TNF-α) and interleukin-6 (IL-6) were estimated using ELISA kits from SinoGeneClon Biotech., following the manufacturer’s protocol. Data were analyzed using a Dynatech Microplate Reader.

The immune response was further evaluated by measuring serum immunoglobulin levels using rabbit polyclonal antibodies against IgE and IgG (COBAS, U.S.A.), with a reagent kit from Elecsys and Precicontrol, U.S.A. Antioxidant indices were determined by measuring serum levels of malondialdehyde (MDA) as an oxidative marker using a kit obtained from Biodiagnostic in Dokki, Giza, Egypt. M.D.A. levels were assessed according to the manufacturer’s instructions.

### Behavioral assessment using the open field test

Behavioral testing was conducted over five minutes to assess exploratory and general activity, with parameters scored via video tracking. The movement of each tested animal in the peripheral and central zones of a 42 × 42 × 42 cm PVC open-field enclosure was measured. Rabbits underwent repeated testing to evaluate drug acclimation/sensitization^52^. The time was adjusted using a separate handheld timer. The present study aimed to evaluate the extent to which scabies infection affects rabbits’ behavior and locomotor activity. Additionally, it examined the possible effect of using IVR gel as a topical treatment of mange on rabbit behavior. Results were compared with animal groups that received the commercial IVR injection as a traditional treatment of scabies. Finally, all results were compared with a negative control group confirmed to be free from mange infection or any other parasitic infestation.

Methods and behavioral parameters: behavioral parameters assessed included total displacement, central displacement, movement, exploration, escape attempts, hops, standing still, and rearing as described in open field studies with rabbits^53,54^. Data was collected across all the pre-treatment and post-treatment phases. All animals within each cohort were handled consistently to minimize stress factors.

### Histopathological and morphometric examination

For histological examination, samples were obtained from all tested groups of rabbits to determine the histological architecture of the skin in the control, infected, and treated groups. Skin samples were collected from the ears and noses of the tested rabbits. Tissues were fixed in 10% formalin, dehydrated in ethanol, cleared in methyl benzoate, and embedded in paraffin wax. Sections (4–6 μm thick) were cut using a microtome and stained with hematoxylin and eosin (H&E)^55^ Stained sections were examined and photographed using an Olympus BX51 microscope equipped with a DP72 camera.

For histomorphometric analysis, the keratin thickness and the combined thickness of the stratum spinosum and stratum granulosum were measured using ImageJ and analyzed using SPSS 26.0. Data are presented as mean ± SD.

### Statistical analysis

Data are presented as mean ± SD for in vitro results and mean ± SD for in vivo results. Normality (Shapiro–Wilk test) and homogeneity of variance (Levene’s test) were assessed prior to analysis to guide parametric/non-parametric test selection. For in vitro analyses (IBM SPSS v26.0), probit regression was estimated to calculate Lethal concentration 50/Lethal time 50 (LC_50_/LT_50_) values. Kaplan–Meier survival curves compared treatments via Log-rank test (χ², df = 1). Mortality rates across concentrations used two-way ANOVA (parametric, assumptions met) with Bonferroni post-hoc correction. While for in vivo analyses (GraphPad Prism v6.07), Kruskal–Wallis one-way ANOVA test (non-parametric) with Dunn’s/Tukey’s multiple comparisons test assessed group differences (*n* = 6/group, pre-planned comparisons). *P.* value < 0.05 was considered statistically significant.

## Results

### Fourier transform infrared (FT-IR) studies

**Fig. 1:**
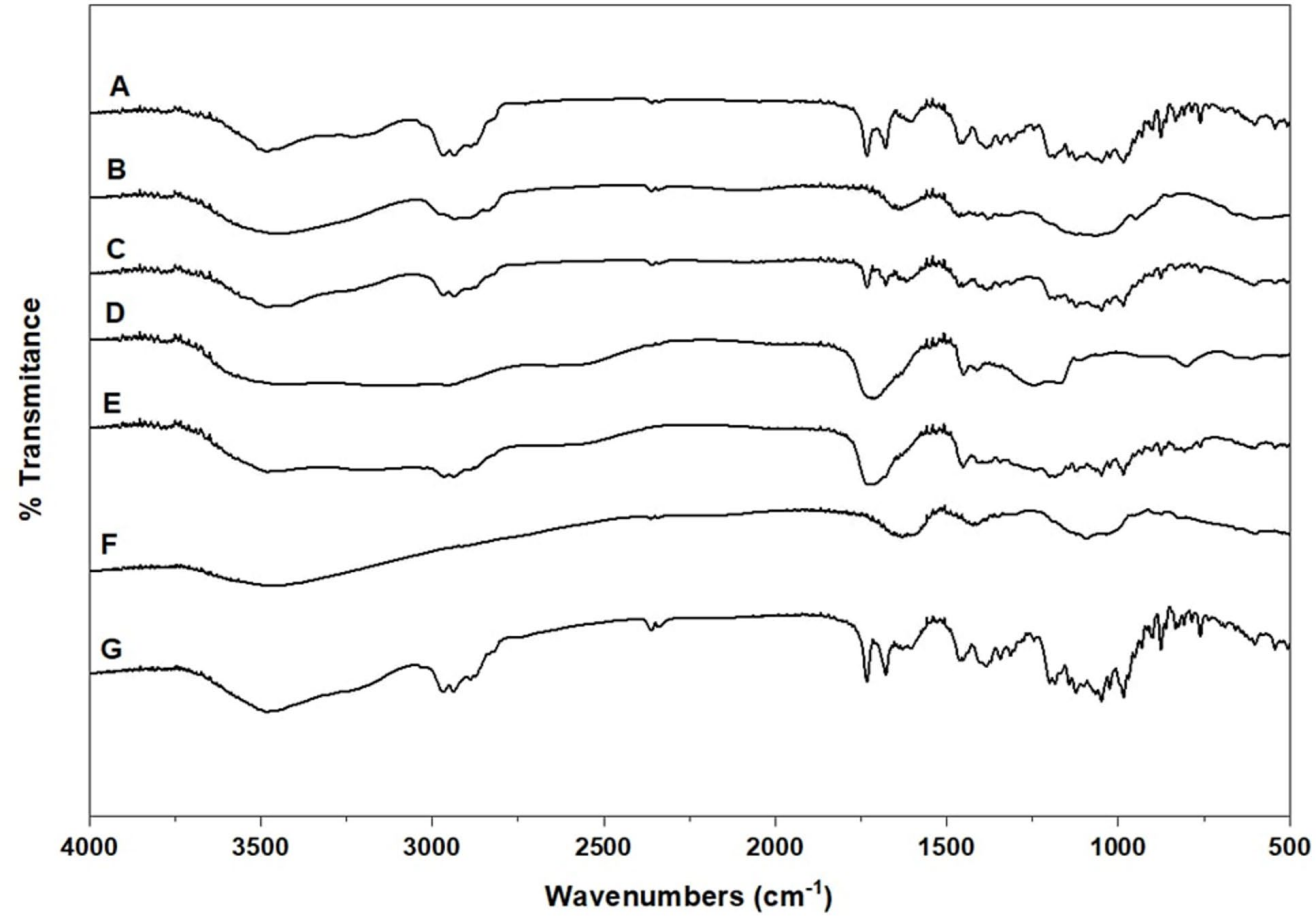
FT-IR Spectra of Ivermectin alone and its physical mixtures with the used polymers (1:1w/w); (**A**) Ivermectin alone. (**B**) HPMC alone. (**C**) Ivermectin: HPMC; (**D**) Carbopol 934 alone. (**E**) Ivermectin: Carbopol 934; (**F**): Na alginate alone. (**G**) Ivermectin: Na alginate.

Figure (1) Shows the FT-IR spectra of Ivermectin alone, the studied polymers alone and the physical mixtures of ivermectin with the studied polymers (1:1 w/w). FT-IR spectrum of Ivermectin alone (Trace A) shows a major characteristic bands at ~ 1732 cm⁻¹ due to lactone (C = O), ~ 3450–3300 cm⁻¹ due to hydroxyl group, ~ 2950 cm⁻¹ due to alkyl group, and 1250–1150 cm⁻¹ due to C–O stretching, corresponding to its multi-functional macrocyclic structure. The principal peaks of ivermectin were observed in the spectra of its physical mixtures with the used polymers^58^.The spectra of the physical mixture of ivermectin with the studied excipients (Fig. 1, traces C, E and G) show the same bands of the drug superimposed with the bands of the studied excipients. The appearance of the same characteristic bands of the drug with slight reduction in the intensity of some bands may be attributed to the dilution effect of the excipient in the studied physical mixture. In addition, the IR-absorption spectra of these physical mixtures show the same characteristic bands of ivermectin without frequency shift, which indicated the absence of chemical interactions between the drug and the used excipients.

### Ivermectin gel characterization

#### Organoleptic properties

All the formulated gels exhibited a transparent, homogeneous appearance with smooth consistency and no visible lumps, as detailed in Table 3. The freshly prepared ivermectin gels displayed colors within the acceptable range.

**Table 3:** Physical characteristics, drug content, pH, and spreadability of the formulated ivermectin gel.

Formula No.	pH value	Drug content	Color	Homogeneity	Transparency	Spread circle diameter Average (cm) ± SD
V1	6.8 ± 0.10	99.5 ± 0.19	White	All gel formulas were homogenous.	All gel formulas are translucent.	3.14 ± 0.03
V2	6.4 ± 0.13	99.8 ± 0.13				2.15 ± 0.01
V3	7.3 ± 0.12	97.2 ± 0.12				2.05 ± 0.01
V4	7.4 ± 0.14	98.8 ± 0.15				1.12 ± 0.02
V5	6.7 ± 0.13	97.5 ± 0.12				3.04 ± 0.10
V6	6.5 ± 0.12	97.8 ± 0.15				1.15 ± 0.06

#### Drug content determination

As presented in Table 3, the drug content of the various ivermectin topical gels ranged from 97.2 ± 0.12% to 99.8 ± 0.13% w/v. This high drug content was deemed satisfactory, confirming the effectiveness of the preparation method.

#### PH measurements

The measurements revealed that the pH of the prepared formulations fell within a range of 6.4 ± 0.13 to 7.4 ± 0.14.

#### Spreadability

Table 3 shows that the developed ivermectin gels exhibited relatively good spreadability. The results were consistent with their viscosity values.

#### Rheological studies

Table 4 shows the viscosities of the prepared gels containing 1% w/v IVR. The viscosities of the prepared IVR gel bases are arranged in the following order: Na alginate ˃ Carbopol 934 > HPMC. The lowest viscosity is obtained with HPMC gel (3% w/v) and the highest one with Na alginate gel (8% w/v), as depicted in Table 3.

**Table 4:** Viscosities of the prepared 1% w/v Ivermectin gels at 37 °C.

Formula No.	Polymer	Shear rate (rpm)							
		10	20	30	40	50	60	70	80
V1	**3% HPMC**	40,300	40,200	40,000	35,200	34,000	33,225	32,200	30,123
V2	**6% HPMC**	40,516	40,500	40,377	37,300	37,000	35,576	34,000	32,576
V3	**0.5% Carb.934P**	50,000	58,897	48,567	38,100	36,789	26,500	24,786	22,500
V4	**1% Carb.934P**	53,300	52,897	40,987	40,000	39,876	38,567	28,500	25,700
V5	**5% Na alginate**	43,000	53,000	51,789	51,500	51,000	50,465	48,546	48,100
V6	**8% Na alginate**	55,000	54,980	55,000	53,080	51,987	51,090	50,767	50,000

#### In vitro release of Ivermectin from the prepared gels

**Fig. 2:**
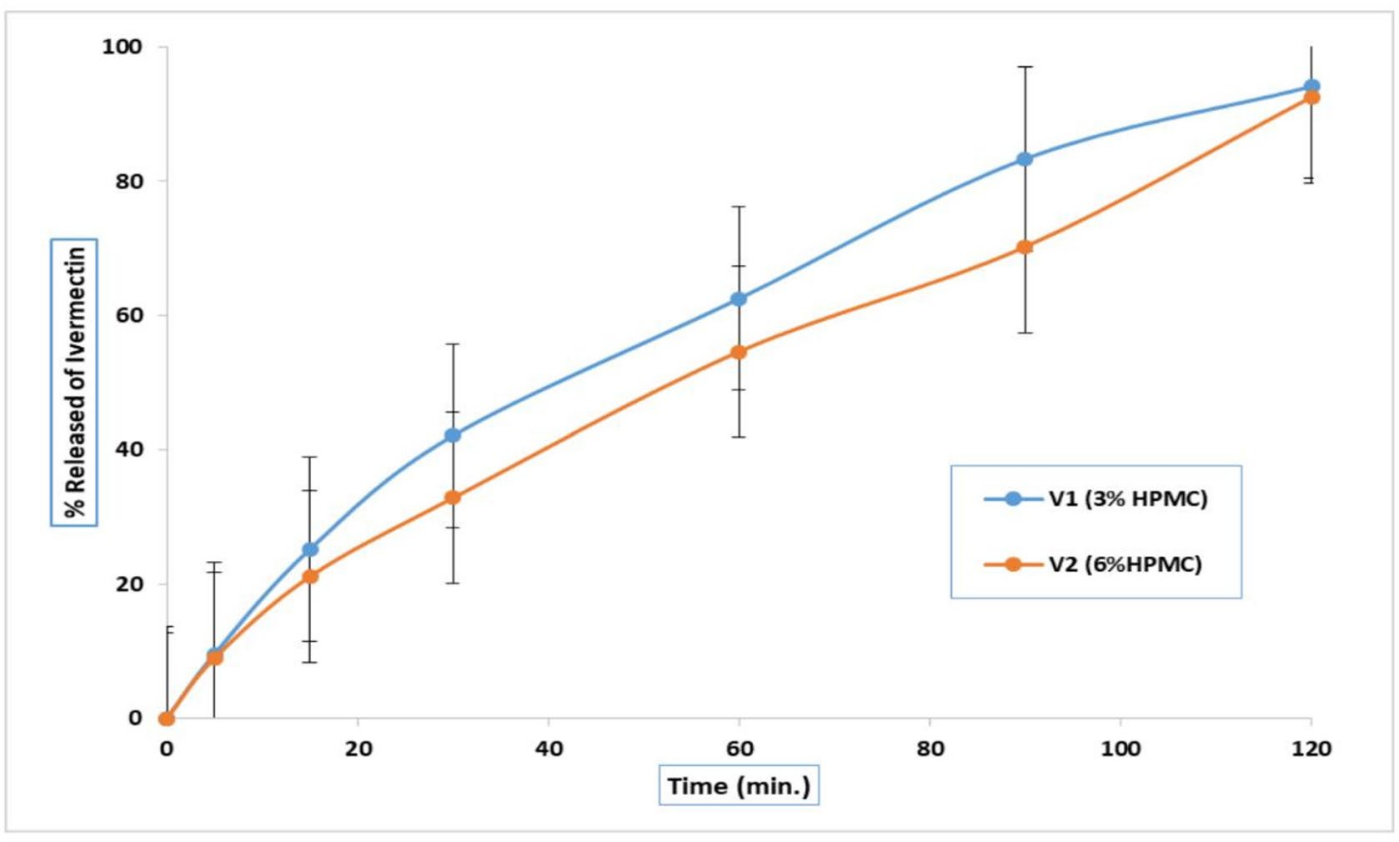
Release profiles of Ivermectin from the prepared gels using HPMC (3 &6% w/v) as a gelling base.

**Fig. 3:**
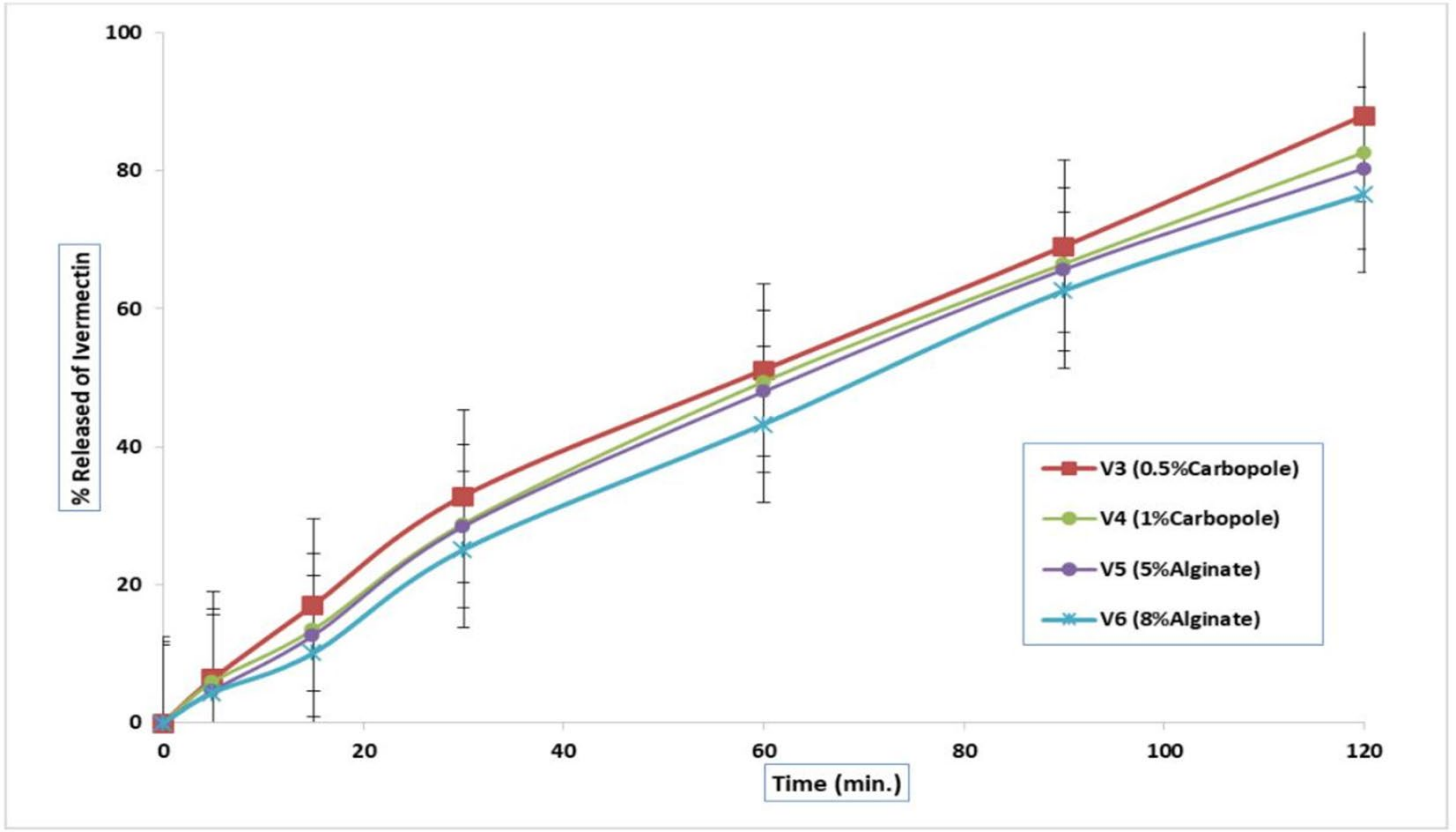
Release profiles of Ivermectin from the prepared gels using Carbopol (0.5 &1% w/v) and Na alginate (5 &8% w/v) as gelling bases.

The release of ivermectin from the different gel bases was evaluated at pH 6.8 over 2 h and is presented in Figs. 2 and 3. The polymers used in the formulations include hydroxypropyl methylcellulose (HPMC), Carbopol 934, and sodium alginate (Na-alginate). Based on gel type, the drug release percentage followed this descending order: HPMC (3% w/v) > HPMC (6% w/v) > Carbopol (0.5% w/v) > Carbopol (1% w/v) > Na-alginate (8% w/v) > Na-alginate (5% w/v), as shown in Fig. 2. Among these formulations, V1, which contained HPMC (3% w/v) as the gelling agent, exhibited the highest drug release (94.13 ± 0.01%) within 2 h.

The release profiles of IVR (1% w/v) from gel formulations (V3–V6) are shown in Fig. 3, with the formulations made using Carbopol 934 (0.5% and 1% w/v) and Na-alginate (5% and 8% w/v) as gel bases at pH 6.8. The drug release rate from these polymers was as follows: Carbopol (0.5% w/v) > Carbopol (1% w/v) > Na-alginate (5% w/v) > Na-alginate (8% w/v).

#### Analysis of the in vitro release profile of ivermectin

The kinetic analysis of ivermectin release from the developed gels is presented in Table 5. Data from the in vitro drug release experiments were matched to several kinetic models, specifically zero-order, first-order, and Higuchi diffusion models. Findings showed that all the tested gels conformed to the Higuchi model of drug release, with higher correlation coefficient (r²) values compared with those calculated for zero-order and first-order kinetics. V1 was selected for in-vivo study due to its highest drug release (~ 94%) and diffusion-controlled mechanism (Higuchi model), indicating efficient drug transport. Its relatively lower viscosity may further enhance skin permeation compared to more viscous formulations, which may retain the drug but limit diffusion. This balance supports improved in vivo therapeutic performance.

**Table 5:** Kinetic analysis of ivermectin release data from the prepared gels.

Formulae no.	Zero order		First order		Higuchi diffusion model		Best fitted model
	r2	K0	r2	Kf	r2	Kh	
V1	0.9821	0.7204	0.9857	0.0228	0.9991	9.8527	Higuchi Diffusion model
V2	0.9945	0.6709	0.9774	0.0168	0.9966	9.0389	
V3	0.9946	0.6883	0.9756	0.0166	0.9959	9.2667	
V4	0.9947	0.6677	0.9903	0.0143	0.9955	8.9841	
V5	0.9933	0.6586	0.9942	0.0134	0.9965	8.8852	
V6	0.9929	0.6389	0.9943	0.0121	0.9957	8.5689	

**r**: correlation coefficient, **K**_**0**_: zero-order rate constant (µg. min^− 1^), **K**_**f**_: first-order rate constant (min^− 1^) and **k**_**h**_: Higuchi rate constant (µg. min^− 1/2^).

### In vitro mortality evaluation

**Fig. 4:**
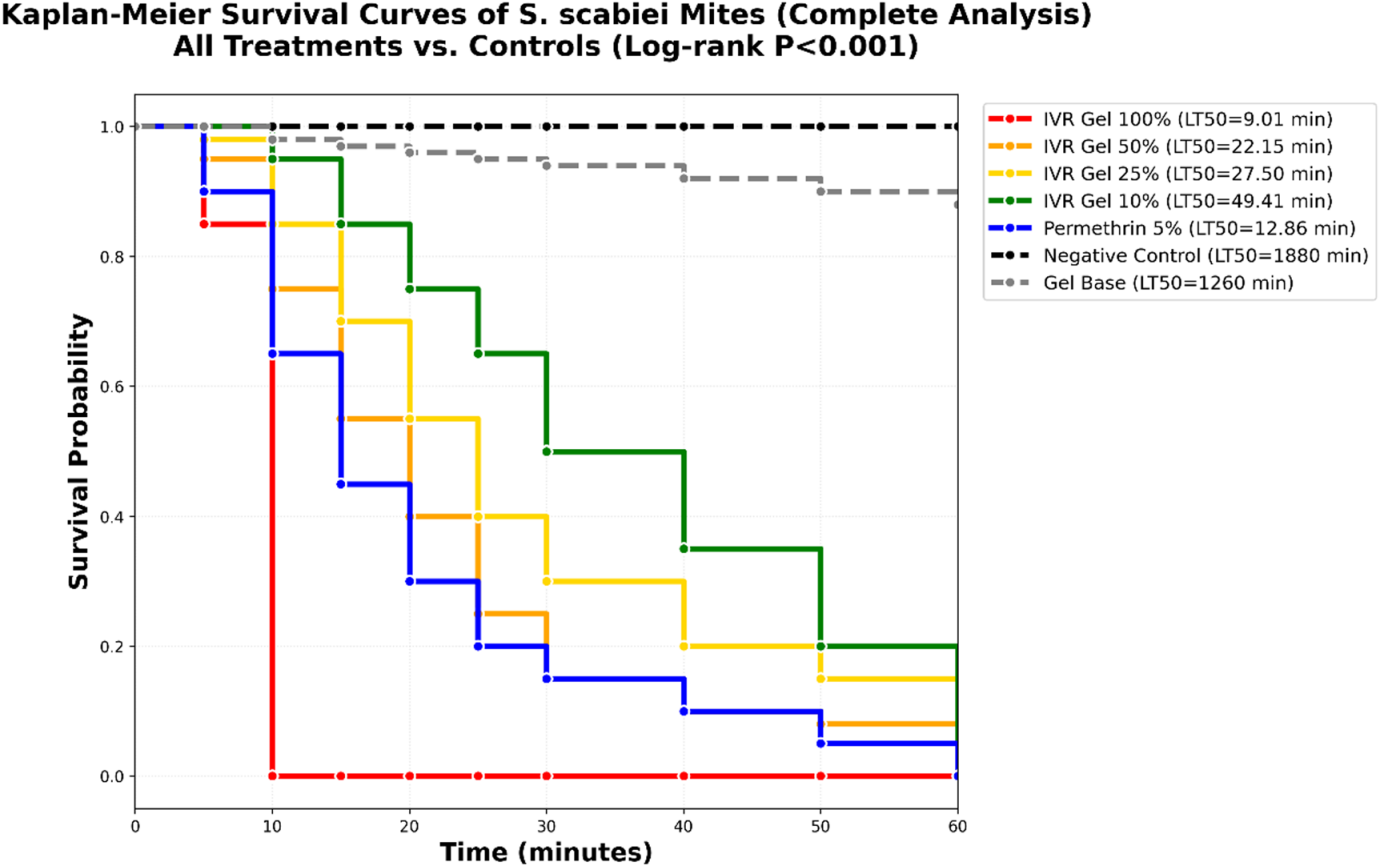
Survival curves of *Sarcoptic scabiei* mites exposed to different drug concentrations *in vitro.* IVR (Ivermectin), Permethrin (positive control).

The aim of this in vitro study was to assess the impact of IVR hydrogel preparation on mites at various concentrations and to determine the optimal concentration for the subsequent in vivo bioassay. The mortality rates at different concentrations of IVR gel (100%, 50%, 25%, and 1%) were evaluated. At a 100% concentration, IVR hydrogel resulted in 100% mortality of *Sarcoptes* mites within 10 min (LT_50_ = 9.01 ± 2.4 min, *P* < 0.001 vs. controls; Table 6). This concentration was selected for in vivo application based on its high efficacy. A dose-response relationship was observed, as mortality rates of adult mites decreased with lower gel concentrations. At 10% concentration, 100% mortality was achieved by 60 min (LT_50_ = 49.41 ± 9.7 min). The LC_50_ values for IVR gel were 48.22%, 27.89%, and 13.37% at 5, 15, and 30 min of treatment, respectively (Table 7). The survival curve demonstrated that all concentrations were lethal within 60 min of the experiment (Fig. 4). LC_50_ values at 60 min could not be determined because all concentrations resulted in 100% mortality.

**Table 6:** Median lethal times (LT_50_) of different IVR hydrogel concentrations.

Tested drug	LT_50_ ± SD (min)	Ⅹ_2_	*P*-value
Negative control (paraffin oil)	1880 ± 45.6	158.59	< 0.001
Gel Base	1260 ± 38.1	68.94	< 0.001
Ivermectin Gel 100%	9.01 ± 2.4	42.59	< 0.001
Ivermectin Gel 50%	22.15 ± 5.1	72.34	< 0.001
Ivermectin Gel 25%	27.50 ± 6.3	76.95	< 0.001
Ivermectin Gel 10%	49.41 ± 9.7	94.25	< 0.001
Positive control (Permethrin 5%)	12.86 ± 3.4	54.35	< 0.001

**Table 7:** LC_50_ values of the ivermectin hydrogel required to kill 50% of *Sarcoptes* mites at different exposure times.

Time (min)		5	15	30	60
LC_50_		48.22	27.89	13.37	-
Confidence limits	**Lower**	37.16	15.74	0	-
	**Upper**	62.64	36.43	23.1	-

### Scanning electron microscopy findings

**Fig. 5:**
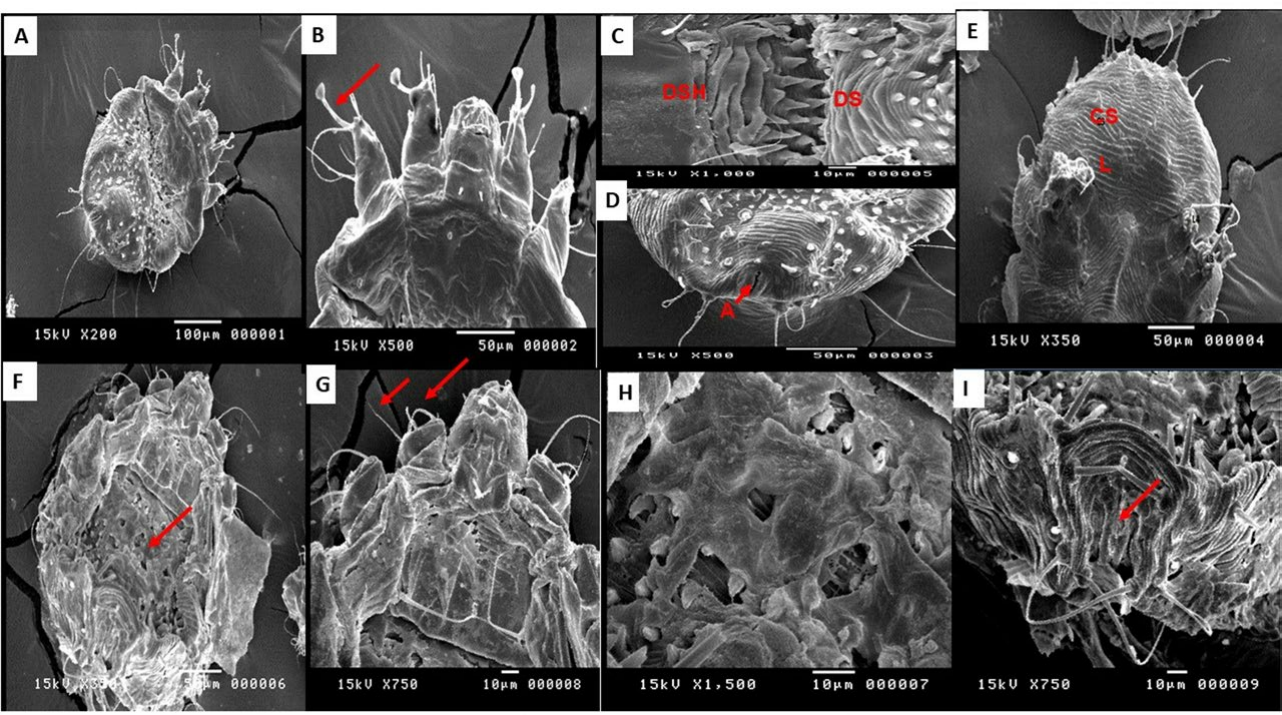
The photomicrograph shows the ultrastructural changes of *Sarcoptes* mites at a 10% concentration of IVR gel in comparison to control mites. (**A-D**): the normal ultrastructure of the negative control *S. scabiei* adult stage is depicted, showing a normal dorsal surface and cuticular spines (**A**), normal tarsus, empodium, and suckers (**B**), intact dorsal shield (D.S.H.) and dorsal setae (**C**), and an intact anal opening and cuticle (**D**). E: ultrastructural findings of Adult *Sarcoptes* mite treated with gel base as a control showing preserved cuticular striations (CS) with a smoother appearance and somewhat folded legs (L). F-I: showing mite’s cuticular changes in contact with IVR gel at 10% concentration, revealing sloughed dorsal shield and loss of cuticular spines (**F**), falling off empodium and suckers from the tarsus (**G**), and deformities in the anus, dorsal shield, and cuticle (**H & I**).

In this study, SEM was used to examine the ultrastructural changes in *S. scabiei* adult mites treated with varying concentrations of IVR gel compared with the controls. Notably, the 10% concentration demonstrated the initial effects of the IVR gel on adult mites, including complete loss of suckers and empodium, sloughing of dorsal lamellar spines, and deformation and sloughing in the region of the anus **(**Fig. 5).

**Fig. 6:**
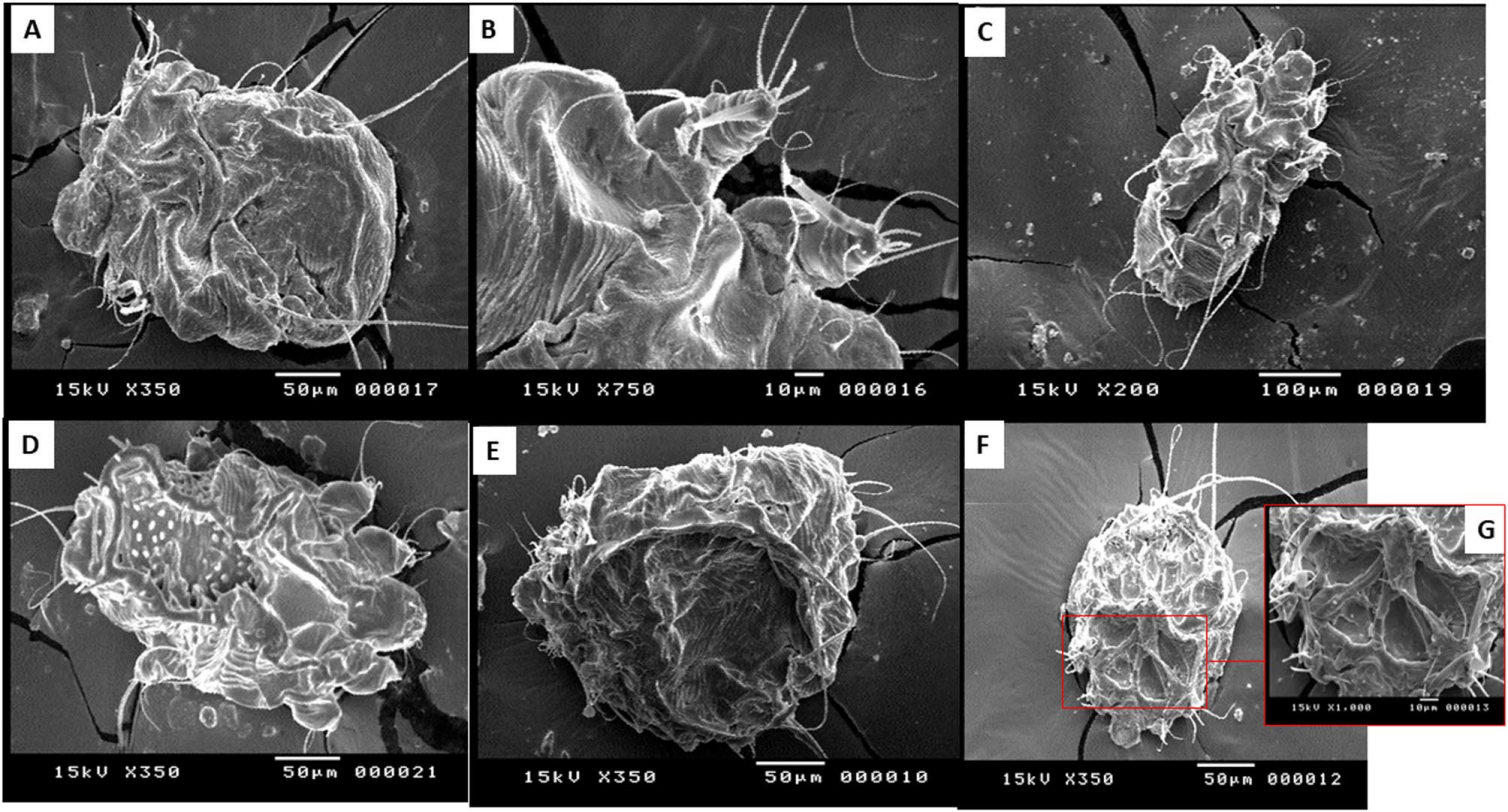
Photomicrograph showing the ultrastructural changes in *Sarcoptes* mites at different IVR gel concentrations. At 25% concentration (**A & B**), the adult cuticle appears dry and wrinkled, whereas the anterior stalked empodium shows the loss of suckers. At 50% concentration (**C & D**), severe dryness, abnormalities, and folded cuticles are observed. At 100% concentration (**E-G**), a significant loss of cuticle material is observed from the mite body.

At 25% concentration, the front legs and suckers detached from their usual position, causing the entire body to become dry and shrivelled, resulting in abnormal wrinkling of the cuticle. At 50% concentration, abnormalities were observed in the front legs, and the entire body exhibited severe contortions, folding, and narrowing. Direct exposure to the gel formulation at a 100% concentration resulted in complete loss of the cuticle in both the front and back regions of the body accompanied by marked deformity (Fig. 6).

### Serological and biochemical findings

The study found that rabbits infested with *Sarcoptes* mites showed a substantial rise in specific IgG and IgE serum levels, in addition to increased levels of inflammatory markers like IL-6 and TNF-α, as well as a considerable increase in the antioxidant indices of MDA compared with the control group. However, IVR hydrogel and commercial IVR treatments showed non-significant changes in serum IgG and IgE levels vs. infected controls (*P* > 0.05; Table 8), whereas there was a decrease in the anti-inflammatory markers of TNF-α and IL-6 (*P* < 0.01 and *P* < 0.001, respectively) compared with the infected untreated group. Furthermore, IVR hydrogel reduced serum MDA levels compared to infected controls (e.g., 375.3 ± 3.05 nmol/mL vs. 410.3 ± 4.51 nmol/mL, *P* < 0.001; Table 8), whereas commercial IVR increased MDA (420.5 ± 1.80 nmol/mL, *P* < 0.05 vs. infected group). Moreover, there are nonsignificant changes between IVR hydrogel and commercial IVR injection-treated groups along the whole measured blood parameters except MDA, showing a significant increase in the commercial one (*P* < 0.001) (Table 8).

**Table 8:** Modulation of inflammatory markers, immune response, and oxidative stress by IVR hydrogel preparation in rabbits infested with *S. Scabiei.*

	Control group	Infected untreated group	IVR- gel-treated group	IVR injection-treated group	*P*- value
IgG (Mmol/L)	97.33 ± 0.58^b^	114.0 ± 4.36^a^	106.7 ± 0.58^a^	106.8 ± 1.26 ^a^	0.002
IgE (IU/ml)	12.33 ± 1.53^b^	19.00 ± 1.00^a^	16.33 ± 0.58^a^	16.67 ± 1.26 ^a^	0.007
IL-6 (ng/L)	123.5 ± 1.85^a^	141.7 ± 1.30^b^	129.4 ± 1.15^c^	129.1 ± 1.15 ^c^	0.001
TNF-alpha (ng/L)	84.60 ± 0.52^a^	93.40 ± 1.92^b^	87.91 ± 0.18^c^	88.57 ± 1.10 ^c^	0.001
MDA (nMol/ml)	211.7 ± 4.51^a^	410.3 ± 4.51^b^	375.3 ± 3.05^c^	420.5 ± 1.80 ^d^	0.001

IgG: immunoglobulin G; IgE: immunoglobulin E; IL-6: interleukin 6; TNF-alpha: tumor necrosis factor alpha; MDA: malondialdehyde. Data are presented as Mean ± SD Values in the same row, followed by different superscripts (a, b, c, d) indicating statistical significance (*P* < 0.05).

### Clinical evaluation

#### Before treatment

**Fig. 7:**
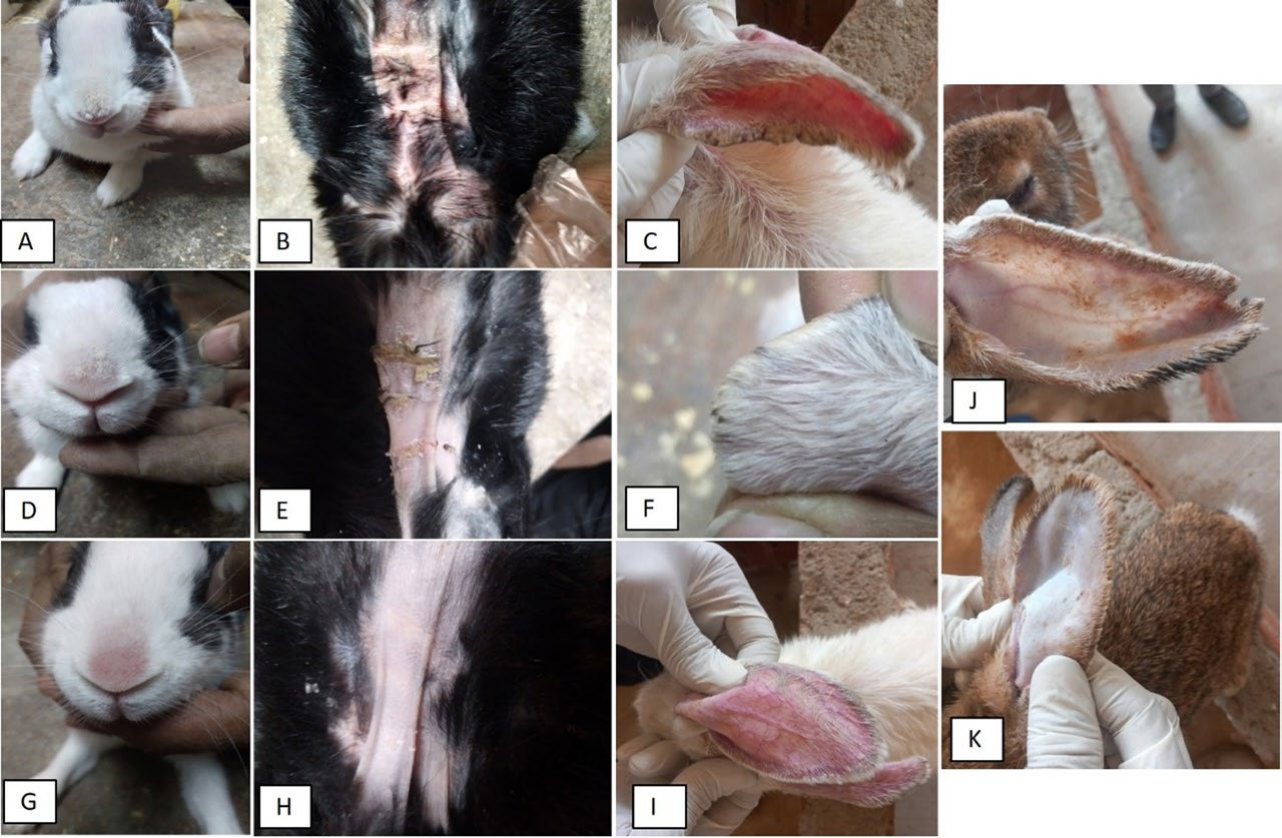
Clinical evaluation of rabbits infested with mites before treatment (**A-C**) and after IVR gel treatment (**D-H**);A: Crusts found on the nasal skin with ruffled fur, B: loss of fur and presence of scales and inflammation in the back of the affected rabbit, C: inflammation and severe crusts on the skin of the ear On the 7th day after treatment, the skin showed signs of skin healing starting with a few crusts in the nose (**D**), back skin (**E**) and ear (**F**).On the 14th day after treatment, the nose appeared normal with no scales and some redness (**G**). The ear has arranged fur with reduced inflammation (**I**). The back has no erythema or scales, but with some parts of alopecia (**H**). (**J**): Macroscopic appearance of rabbit pinna 7 days after initial IVR injection treatment showing persistence of thick, hyperkeratotic crusts with significant alopecia and erythema. K: 14 days post IVR injection treatment showing a decrease in erythema and popular rash, detachment of scabs, and the initiation of partial hair regrowth.

The diseased rabbits included in the study displayed a range of scabies clinical signs, including a lack of appetite, anemia, intense itching, and redness of the skin, and the presence of white, hard, dry crusts on the nose, ears, around the ears, face, legs, around the genital organs, and over the back, with a brownish discoloration. Lesions ranged from moderate to severe (clinical score ranged from 4 to 5) (Fig. 7).

#### After treatment

As shown in Table 9, on the 7th day after IVR gel treatment, a noticeable improvement in clinical scoring of lesions was observed, with only mild lesions remaining. Most of the skin crusts began to fall off, and the lesions began to disappear, leading to the partial recovery of the rabbits (Mean score ± SD = 2.72 ± 0.67) (Figs. 7D-F). By the 14th day post-treatment, all rabbits tested negative for mite infestation via stereomicroscopic examination. There were no clinical signs of weakness, depression, anorexia, or anaemia. The rabbits had significant improvement with resolution of most clinical signs of scabies (*P* < 0.001), such as disappearance of scales and the itching and secondary sores had disappeared entirely (overall mean score = 1.00 ± 0.60) compared with the infected untreated group observations (mean score = 4.78 ± 0.44) (Figs. 7G-I). After 21 days of treatment, the overall condition of the treated rabbits significantly improved, and all skin lesions disappeared. Any lesions resulting from scratching had healed across the entire body surface, including the face, leading to the start of regrowth of fur and the reduction of alopecia (overall mean score = 0.17 ± 0.18, *P* < 0.001).

Regarding IVR injection treatment, during the seven days post 1 st dose of IVR injection, no change in clinical signs and lesions was observed; the mean lesion score was 4.08 ± 0.21 (*P* = 0.187). On the 14th day post-treatment (7 days after the second dose of IVR injection), signs of healing began to appear with partial regrowth of fur, less erythema of skin, decreased appearance of scabs, and decreased alopecia with few mites detected on examination, with a mean lesion score of 3.08 ± 0.67(*P* < 0.01).

**Table 9:** Clinical scoring of the curative effect of IVR gel on rabbits infected with *S. scabiei* for 21 days post-treatment.

Animal group	Site	Day 0	Day 7	Day 14	Day 21
Infected untreated control	Nose	4.00 ± 0.00	4.17 ± 0.41	4.33 ± 0.52	4.40 ± 0.55 *
	Back	5.00 ± 0.00	5.00 ± 0.00	5.00 ± 0.00	5.00 ± 0.00 *
	Ears	5.00 ± 0.00	5.00 ± 0.00	5.00 ± 0.00	5.00 ± 0.00 *
	Overall	4.67 ± 0.52	4.72 ± 0.47	4.78 ± 0.44	4.80 ± 0.45 **
IVR gel-treated group	Nose	4.00 ± 0.00	3.00 ± 0.63	1.83 ± 0.75	0.50 ± 0.55
	Back	5.00 ± 0.00	3.17 ± 0.75	1.00 ± 0.63	0.00 ± 0.00
	Ears	5.00 ± 0.00	2.00 ± 0.63	0.17 ± 0.41	0.00 ± 0.00
	Overall	4.67 ± 0.52	2.72 ± 0.67	1.00 ± 0.60	0.17 ± 0.18
IVR Inj. treated group	Nose	4.00 ± 0.00	4.00 ± 0.00	3.17 ± 0.75	2.83 ± 0.75
	Ears	5.00 ± 0.00	4.17 ± 0.41	3.00 ± 0.63	2.33 ± 0.52
	Overall	4.50 ± 0.55	4.08 ± 0.21	3.08 ± 0.67	2.58 ± 0.62
*F* value		0.14	85.48	178.67	220.69
*P* value		0.87	< 0.001*	< 0.001*	< 0.001*

*20% of infected, untreated animals died by Day 21. Scores represent the mean of the remaining animals.

**Overall score is the mean of the scores from all body sites for each group.

### Behavioral assessment

Exploratory behavioral observations indicated that *S. scabiei*-infected rabbits exhibited a pattern of reduced activity, with a lower total movement count compared with the uninfected control group. Furthermore, the duration of time devoted to movement was significantly shorter in the infected group than in the uninfected control group (*P* < 0.05), consistent with sickness behavior profiles. Regarding treatment groups, rabbits administered IVR gel or IVR injection displayed significantly lower total displacement, movement, and hopping frequencies compared with the uninfected control group (*P* < 0.05). In terms of spatial use, the time spent in central displacement did not differ significantly among the groups. Conversely, immobility (time spent standing still) was significantly prolonged in the infected untreated group compared with both treated groups (IVR gel or injection). Overall, healthy rabbits displayed patterns of higher movement, hops, and exploratory behavior than both the infected and treated rabbit groups. No significant differences were observed in central movements, escape attempts, or rearing behaviors between the healthy control, infected, and treated groups **(**Table 10**).**

**Table 10:** Open field test for testing animal locomotion and anxiety after IVR gel formulation treatment.

	Infected untreated group	IVR gel-treated group	IVR injection-treated group	Negative control	*P*-value
Total displacement *t*	8.66 ± 4.40^b^	7.00 ± 2.51^b^	7.3 ± 3.12^b^	23.66 ± 1.45^a^	0.001
Central displacement	1.33 ± 0.66	1.33 ± 0.66	1.34 ± 0.56	1.00 ± 0.33	0.999
Movement	3.94 ± 1.29^b^	7.68 ± 0.61^b^	7.9 ± 0.52 ^b^	14.54 ± 0.52^a^	0.034
Exploration	51.33 ± 4.97^b^	51.66 ± 10.17^b^	51.74 ± 7.12 ^b^	59.83 ± 0.37^a^	0.013
Escape attempts	2.66 ± 0.33	2.33 ± 1.85	2.54 ± 1.43	5.00 ± 1.15	0.950
Hops	9.00 ± 3.21^b^	7.00 ± 2.08^b^	7.4 ± 2.31 ^b^	20.50 ± 2.59^a^	0.001
Standing stills	43.73 ± 14.12 ^b^	36.39 ± 9.89^c^	37.35 ± 10.67^c^	27.86 ± 7.27^a^	0.013
Rearing	3.33 ± 1.76	1.33 ± 0.88	1.45 ± 1.2	4.00 ± 1.73	0.983

^a, b^ Different superscript letters indicate significant differences (*P* ≤ 0.05). IVR= ivermectin. Negative control= uninfected animal group.

### Histopathological and histomorphometry findings

**Fig. 8:**
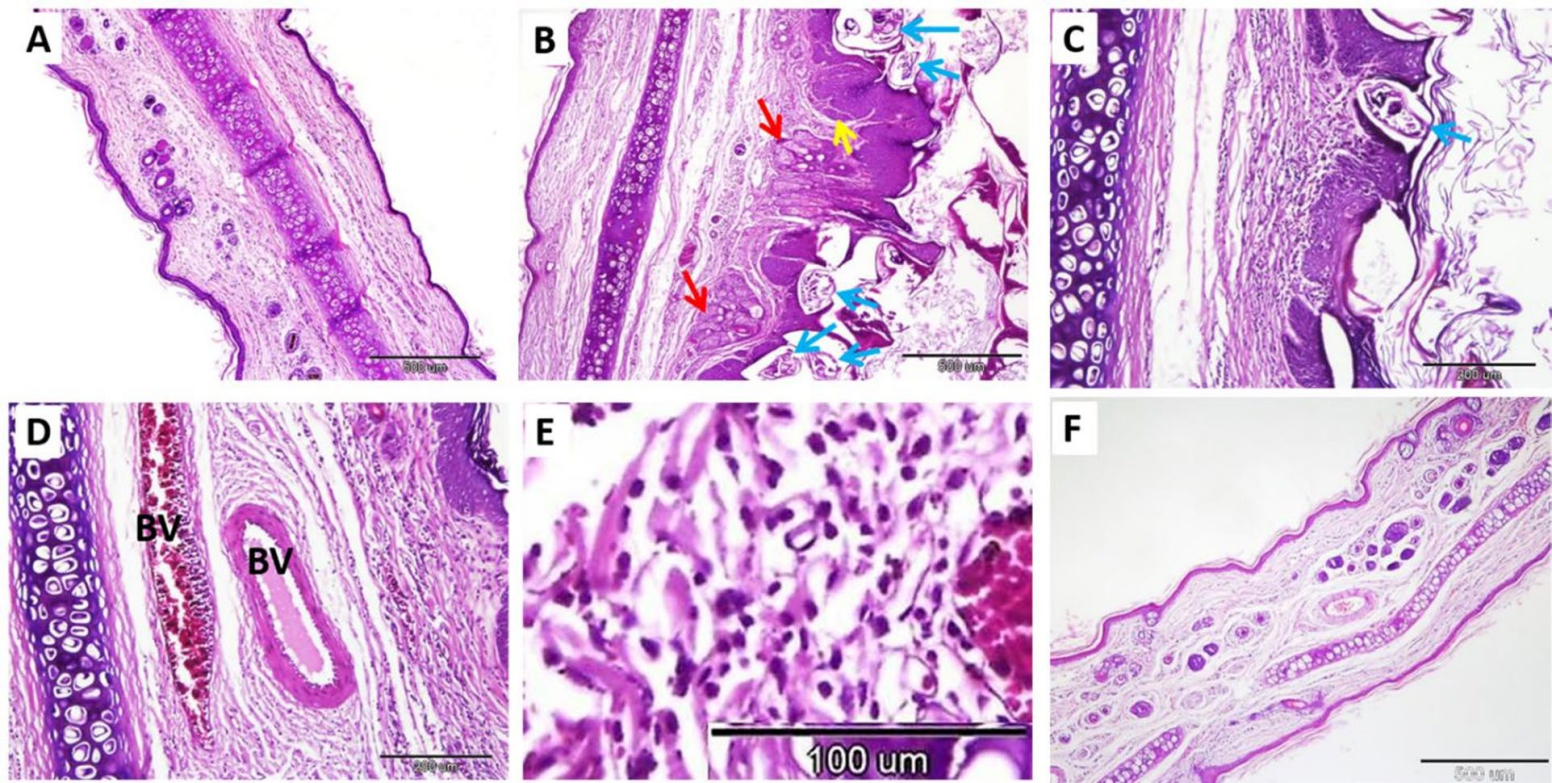
Photomicrographs of the rabbit ear skin in the control negative group (**A**), infected untreated group (**B-E**), and treated group (**F**). A: Normal skin architecture. (**B**): mites (blue arrows), sebaceous glands (red arrows), and rete ridge hypertrophy (yellow arrows). (**C**): burrowing mites (blue arrow). (**D**): Inflammatory cell infiltration fills the dermal vessels (B.V.). E: dermis inflammatory cell infiltration. (**F**): normal non-infested ear skin of the treated group stained with hematoxylin and eosin. Scale bar (**A**,** B**, and F = 500 μm), (**C and D** = 200 μm), and (**E** = 100 μm).

**Fig. 9:**
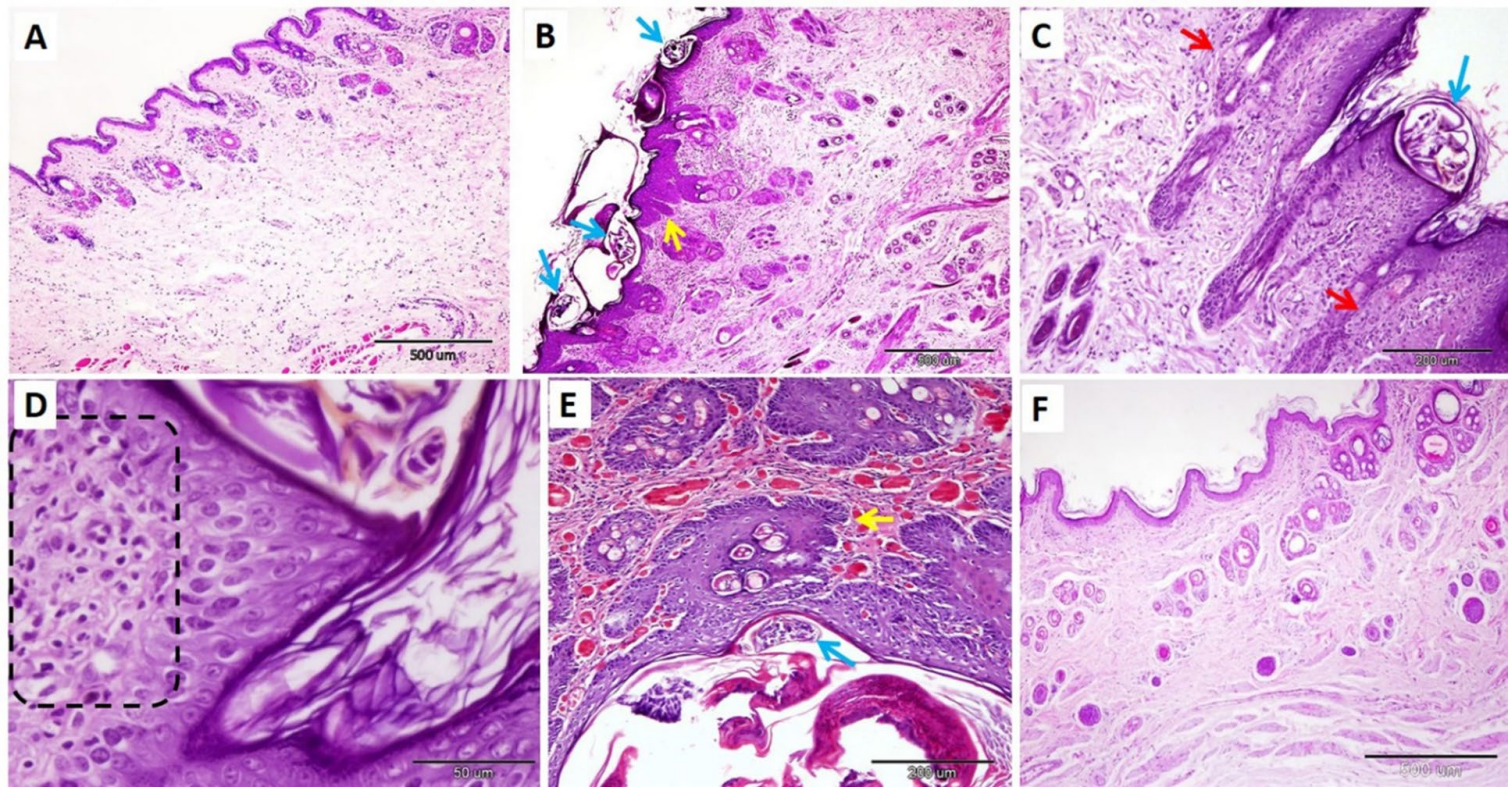
Photomicrographs of the rabbit nose skin in the control negative group (**A**), infected untreated group (**B- D**), IVR injection treated group (**E**), and IVR gel treated group (**F**). A: Normal nose skin architecture. B: mites (blue arrows) and rete ridge hypertrophy (yellow arrows). (**C**): mites (blue arrow) and sebaceous glands (red arrows). (**D**): inflammatory cell infiltration on the dermis (dashed rectangle). (**E**): mite (blue arrow) and rete ridge hypertrophy (yellow arrow). (**F**): Normally, non-infested nose skin of the treated group stained using H&E. Scale bar (**A**,** B**, and **F** = 500 μm), (**C & E** = 200 μm), and (**D** = 50 μm).

Histological examination of the skin of the ear and nose in the negative control group revealed normal skin architecture, including a healthy epidermis and dermis (Figs. 8A and 9A). In the ear, there was an observed thickening of the keratin layer by approximately 11.47 ± 2.15 μm and a thickening of the stratum spinosum and stratum granulosum by nearly 20.90 ± 1.40 μm. Similarly, the nose showed an average thickening of the keratin layer of 11.34 ± 2.59 μm and a thickening of the stratum spinosum and stratum granulosum of 23.79 ± 3.56 μm. (Table 11).

The skin of infected animals exhibited various dermal and epidermal histopathological changes. The epidermis showed hyperkeratosis, hypogranulosis, acanthosis, and hypertrophy of the rete ridges, along with the presence of epidermal tunnels. The dermis exhibited sebaceous gland hyperplasia and hypertrophy, inflammatory cell infiltration in the dermal vessels and epidermis, and oedema characterized by wide interstitial tissue and vein dilatation. Mites (2–5 mites per section) were observed invading the stratum corneum and spinosum, as well as positioned deeply near the dermis and the duct of sebaceous glands (Figs. 8B-E and 9B-D). Additionally, there was a significant increase in the thickness of the keratin layer and stratum spinosum, and stratum granulosum in both the ear and nose (Table 11).

**Table 11:** Comparative analysis of the thickness of the epidermal skin layers among the different animal groups.

Item	Groups				*P*-value
	Negative control	Infected untreated control	IVR injection-treated group	IVR gel-treated group	
Thickness of K (ear)	11.47 ± 2.15 ^b^	58.70 ± 5.65 ^a^	-	22.56 ± 3.07 ^b^	0.001
Thickness of K (nose)	11.34 ± 2.59 ^b^	43.39 ± 4.71 ^a^	16.53 ± 1.23^b^	19.65 ± 2.69 ^b^	0.001
Thickness of S.S. and S.G. (ear)	20.90 ± 1.40 ^b^	57.71 ± 8.13 ^a^	-	16.16 ± 1.20 ^b^	0.001
Thickness of S.S. and S.G. (nose)	23.79 ± 3.56 ^b^	53.27 ± 6.70 ^a^	32.13 ± 0.99^b^	26.49 ± 1.92 ^b^	0.001

Data presented as Mean ± SD. a, b, c Different superscript letters indicate significant differences (*P* ≤ 0.05). K (keratin), S.S. (stratum spinosum), and S.G. (stratum granulosum).

The results of our study revealed that the skin samples in the IVR injection-treated group showed incomplete relief; there is still hypertrophy of the rete ridges, hyperemic vessels, and mites are still observed. However, there was a significant decrease in the thickness of the keratin layer, and stratum spinosum and stratum granulosum in the nose measuring about 16.53 ± 1.23 μm and 32.13 ± 0.99 μm, respectively (Fig. 8E). On the other hand, the IVR gel-treated group exhibited a normal architecture of epidermis and dermis, with no presence of mites. However, hyperemic vessels were observed in the dermis (Figs. 8F, and 9F). Morphometric measurements of the ear and nasal keratin layers were nearly restored to normal, measuring approximately 22.56 ± 3.07 μm and 19.65 ± 2.69 μm, respectively. Additionally, the thickening of the stratum spinosum and stratum granulosum in the ear and nose returned to normal values, measuring about 16.16 ± 1.20 μm and 26.49 ± 1.92 μm, respectively (Table 11). The present study revealed significant growth in the thickness of the keratin layer, stratum spinosum, and stratum granulosum in the infected untreated group compared with the control group. In both treated groups, there was a significant reduction in the thickness of the keratin layer compared with the infected group. Still, the IVR gel-treated group was more effective in restoring the thickness of the stratum spinosum and stratum granulosum. No mites were detected, indicating that treatment with IVR gel is more effective than IVR injection (Table 10).

## Discussion

The most widely used macrocyclic lactone is ivermectin, which exhibits insecticide, acaricide, and anthelmintic properties^56^. This compound is frequently used to treat mange in rabbits and human scabies, particularly in severe forms and in epidemics^57–59^. Though the high effectiveness reported with ivermectin treatment, it is not suitable for children or pregnant women. Besides, some reports revealed that IVR can cause severe toxicity when combined with other medications like methotrexate, cyclosporine, and digoxin. Furthermore, annual treatment failure rates were reported to rise significantly by 0.2% among orally treated patients, prompting recent recommendations for topical preparations as an alternative^60,61^.

The therapeutic effectiveness of topical IVR was compared with that of oral IVR for treating head lice infestation. A previous study found that a single application of a topical IVR solution resulted in a quicker clinical response and higher cure rates compared to a single oral dose. The increased effectiveness of this treatment can be attributed to its direct delivery method and the potentially higher concentrations of the drug that are achieved when applied topically, thus enabling a faster parasiticidal effect^62^. Given the previously described treatment challenges of scabies, this study provided a novel topical IVR gel formulation and evaluated its efficacy in vitro and in vivo in a rabbit model in a trial to offer an effective, well-tolerated, easily applicable topical scabicidal drug.

The in vitro assay demonstrated a significant acaricidal activity of the ivermectin (IVR) hydrogel, with the 100% (w/v) formulation achieving complete mortality of *S. scabiei* mites within 10 min. This rapid effect supported its selection for subsequent in vivo trials. A clear dose–response relationship was also observed. This observed rapidity of action is consistent with the established pharmacodynamic mechanism of IVR, which acts as an agonist of glutamate-gated chloride channels, leading to chloride ion influx, neuronal hyperpolarization, paralysis, and death^63^. Supporting this finding, scanning electron microscopy (SEM) analysis of treated mites revealed corresponding ultrastructural damage, including severe cuticular erosion and body collapse, indicative of rapid physiological breakdown following neuro-muscular paralysis.

Our findings demonstrated notably higher efficacy of the IVR gel formulation compared to earlier studies. A prior in vitro study assessing the acaricidal properties of IVR reported that a 1% IVR solution induced 90% and 100% mortality at 24- and 48-hours post-treatment, respectively^64^. Similarly, Li et al.^65^ observed 100% mortality in adult mites within 2 and 7 h following exposure to IVR concentrations of 0.1 and 0.05 mg/ml in rabbits. Although certain plant extracts and essential oils have also demonstrated rapid acaricidal effects in vitro, their clinical translation is hindered by challenges in standardization, compositional variability, and potential toxicity^66–68^.

The in vivo evaluation in a rabbit model demonstrated that the IVR hydrogel effectively resolved scabies lesions and improved overall health after a three-week follow-up. These results are consistent with the established efficacy profile of topical ivermectin in clinical settings. For instance, prior clinical trials have documented cure rates of 69.3% to 87.5% following a single application of topical ivermectin formulations, with effectiveness often reaching 100% after a second dose or by the end of the second week^69,70^. This timeline aligns with the complete resolution of the lesions observed in our animal model by day 21. Another previous study proved that topical treatment with IVR 0.1% in naturally infected rabbits with mange was the drug of choice, achieving zero mortality of treated animals, disappearance of skin scales, and improvement of all biochemical parameters of rabbits, indicating an improvement in rabbits’ health conditions^74^. However, these preliminary results should be interpreted cautiously due to the study’s limited scale and the use of a single animal model. Further research with larger trials and direct comparisons to standard treatments like permethrin is needed.

The study highlights that *S. scabiei* infection in rabbits induces a pronounced immunological response, characterized by significant elevations in serum IgG, IgE, TNF-α, and IL-6 levels compared to uninfected controls. This pattern aligns with a Type I hypersensitivity reaction, mediated by IgE-driven activation of mast cells, eosinophils, and basophils, which release pro-inflammatory cytokines such as TNF-α, IL-6, IL-4, IL-5, and IL-13. At the tissue level, these cytokines stimulate endothelial cells, keratinocytes, and fibroblasts, further amplifying inflammation through IL-1, IL-6, and TNF-α production^9,71^. The marked increase in IgG and IgE observed in the study reflects an adaptive immune response, likely driven by IL-4/IL-13-mediated B-cell class switching^72^. Additionally, the IgE response is often associated with IgG4 production, though its role in scabies remains unclear and may involve antigen-blocking mechanisms^73,74^. These findings are consistent with previous reports documenting elevated IgA, IgG, and IgM, and increased activity of pro-inflammatory cytokines (e.g., IL-2, IL-6, IL-12, IL-1β, IFN-γ) in *Sarcoptes*-infested subjects compared to controls, underscoring the systemic and local immunopathological impact of infestation^75–78^.

In addition, infested rabbits exhibited marked oxidative stress, with elevated MDA levels indicating lipid peroxidation and cellular damage, consistent with previous reports^79,80^.

Our study demonstrated that both topical and systemic IVR administration significantly reduced key inflammatory markers, specifically TNF-α and IL-6, in treated animals. This finding aligns with previous reports on ivermectin’s anti-inflammatory properties^81^. For the topical IVR hydrogel, a reduction in malondialdehyde (MDA) levels compared to the infected untreated group was observed, suggesting a potential alleviation of oxidative stress associated with scabies infection. This is consistent with reports of improved oxidative stress parameters following ivermectin treatment^82^. Notably, however, serum MDA levels in the gel-treated group remained elevated above normal controls, indicating that treatment mitigated but did not fully resolve the infection-induced oxidative burden.

A notable and complex finding was that systemic IVR resulted in higher MDA levels than the infected untreated group. While this observation points to a formulation-dependent effect, its interpretation requires caution. The elevated MDA may reflect a pharmacodynamic response to the systemic drug or an aspect of the infection’s resolution pathway distinct from topical application. This finding does not necessarily indicate a harmful effect of systemic IVR but highlights a differential impact on oxidative stress markers that requires further investigation to elucidate the underlying mechanism.

The immunomodulatory effects appeared limited in this model, as both IVR treatments did not significantly alter IgG and IgE levels—a result consistent with some previous studies^83^. However, this finding is in contrast with the immunoglobulin modulation of IVR reported in other studies^84,85^. The observed discrepancy in these studies suggests that the immunomodulatory effects of IVR may vary depending on the parasite species and the disease being treated^86,87^.

Our histopathological investigation indicated that *S. scabiei-*infected animals showed different skin histopathological changes, including epidermal thickening, epidermal tunnels, dermal inflammatory reaction, and sebaceous gland hypertrophy. Similar observations have been reported in sarcoptic mange infestation in different animals^9,88,89^. The mite *S. scabiei* completes its life cycle by releasing proteins that degrade the host’s skin. The parasite’s proteins allow it to compromise the skin, leading to a thickened keratin layer and inflammatory cell infiltration. These proteins enable the parasite to compromise the skin, resulting in a thickened keratin layer and inflammatory cell infiltration^8,90–92^.

During burrowing, the parasites cause epidermal tunnels and irritate hosts through saliva, enzymes, feces, and eggs. The body employs a compensatory mechanism by increasing the thickness of the keratin layer^89,93–95^. Sebaceous gland hypertrophy may result from hyperkeratotic crusts that obstruct the gland’s excretory duct^88^. All these previous factors may lead to scratching, inflammation, and immune reactions. Thus, mite infestation may be accompanied by secondary bacterial infection, causing cardiac and renal illnesses^96^.

In the present study, both treated groups showed a significant reduction in the thickness of the keratin layer compared with the infected group. Still, the IVR gel-treated group was more effective in restoring the thickness of the stratum spinosum and stratum granulosum. No mites were detected, indicating that treatment with IVR gel is more effective than IVR injection in this model. This is possible due to rapid and direct action of IVR hydrogel on the *Sarcoptes* skin lesion with anti-inflammatory and antioxidant effects. However, direct comparisons are limited in the present model by differences in administration route, dosing frequency (daily topical vs. intermittent systemic), and local vs. systemic exposure.

On a behavioral basis, the open field test is widely used to assess and analyze locomotion and anxiety-like behaviors in rodents because it is used to evaluate an animal’s general health and well-being^97^. It is considered a valuable tool for assessing disease progression and drug efficacy^97,98^. In this study. Unlike healthy rabbits, both treated animal groups, either with IVR gel or IVR injection, displayed similar changes in locomotive activity, which was quite similar to that of infected animal groups, including both the time spent in different arenas and the number of rears in the open field. Total displacement, time consumed in movement, exploration, and hops were significantly lower in all IVR gel-treated animal groups than the control negative group (*P* ≤ 0.05). Similar results were obtained from the IVR-injected animals. In contrast, standing still was significantly lower in the negative control group than in all other groups. This could be attributed to the fact that healthy rabbits were perceptibly bolder and more likely to show additional locomotive behaviors than infected animals^99^. This finding concurred with Endris and Feki^100^, who confirmed that stressed animals were not entirely healthy, exhibited reduced mobility within their surroundings, and demonstrated lower levels of activity in open-field assessment.

It is noteworthy that measures such as central displacement, escape attempts, and rearing showed no significant differences between infected, treated, or even negative control groups. This may indicate that the anxiety levels in these groups were not severe enough to pose a danger to animal lives, and they acted similarly to healthy animals, except for their movement patterns. According to Salomons et al.^101^, anxiety can be considered a negative emotion without necessarily compromising an animal’s welfare, provided it does not exceed its adaptive capabilities or pose a threat to its well-being. Topical IVR gel-treated animal groups showed no significant difference from the IVR injection group, where both treated groups showed decreased general locomotion compared to the healthy groups. This finding was supported by de Souza Spinosa et al., who reported that administration of IVR may decrease general animal activity^102^. According to our study, using IVR gel as a topical treatment of *S. scabiei* infection did not accentuate any additional effect on the diseased animals’ behavior. Moreover, no significant unusual behaviors were noted throughout the experiment.

Based on these findings, our study demonstrated that topical IVR hydrogel achieved favorable results comparable to systemic IVR in terms of lesion resolution, oxidative stress reduction, and restoration of normal skin architecture without adverse behavioral effects. These results align with prior evidence showing that topical IVR formulations can match the efficacy of standard permethrin therapy when applied correctly. For instance, earlier studies reported that permethrin 5% achieved higher early cure rates (74.8% at one week) compared to oral IVR (30%) and topical IVR (69.3%). However, by two weeks, both permethrin and topical IVR reached near-complete cure rates (99% and 100%, respectively), whereas oral IVR remained lower (63%)^73^. This supports our observation that topical IVR offers rapid and sustained therapeutic benefits, particularly when combined with optimized delivery systems such as hydrogels.

Previous reports on oral IVR emphasizing the need for repeated dosing to achieve full effectiveness^64,73,103^. In contrast, our hydrogel formulation provided consistent improvement without systemic exposure, reducing inflammatory markers (TNF-α, IL-6) and oxidative stress (MDA) comparable to injection therapy, which highlights the potential of topical IVR to minimize systemic side effects while maintaining acaricidal activity. As extensively reviewed in the literature, combination therapy using permethrin along with oral IVR, topical IVR, and synergized pyrethrins demonstrated the strongest evidence for achieving the highest cure rates, the lowest incidence of persistent itching, and the fewest adverse reactions, respectively^77,104^. However, no single treatment excelled in all aspects. Physicians should consider not only the efficacy and safety profiles of medications, but also their ease of administration^104^.

Regarding the IVR gel formulation, freshly prepared IVR gel is characterized by its transparency and homogeneity, and the drug content of various IVR topical gels falls within the accepted pharmacopeial limits, as indicated in Table 2. A higher percentage of the drug content indicates that the drug is uniformly distributed within the specific developed preparations. The viscosity of the prepared IVR formulation is influenced by the type and concentration of the gelling agents used. It can be inferred from Table 3 that the viscosities increased with increasing polymer concentration for all tested polymers. According to Meshali et al.^105^, the increase in gel viscosity with increasing polymer concentration is attributed to the development of a dense network resulting from molecular entanglement or attraction facilitated by hydrogen bonds or van der Waals forces. This aggregation is distinguished by an increase in the viscosity resulting from these forces. At higher speeds, the rate of viscosity decline was significantly greater than that at lower speeds. The molecules in the gel’s three-dimensional network structure are intertwined with the immobilized solvent. As the shear rate increases (increased speed), the network structure breaks down, and the molecules become disentangled and then align in the direction of flow. The presence of molecules decreased the resistance to flow, resulting in lower gel viscosity, primarily due to the release of water from the disrupted network structure.

Furthermore, the viscosities of the prepared IVR gel bases were arranged in the following order: Na alginate ˃ Carbopol 934 > HPMC. Findings from the drug release data indicate that fluctuations in drug release can be due to differences in the structure and viscosity of the gel bases, along with fluctuations in interactions between the drug and polymer. According to the viscosity findings, gels with higher viscosity exhibited lower drug release percentages, whereas those with lower viscosity, such as the HPMC hydrogel base, demonstrated higher drug release^106^. Concerning the gel type, it showed a decrease in the percentage of the drug released from the prepared gels in the following order: HPMC (3%w/v) > HPMC (6%w/v) > Carbopol (0.5%w/v) > Carbopol (1%w/v) > Na alginate (8%w/v) > Na alginate (5%w/v), as described in Fig. (2). As the concentration of HPMC increases, drug release decreases. This can be attributed to the higher HPMC content, which increased gel viscosity, thereby reducing the penetration rate of the dissolution medium.

Furthermore, the rate at which the drug is released from these polymers decreases in the following sequence: Carbopol (0.5%w/v) > Carbopol (1%w/v) > Na alginate (5%w/v) > Na alginate (8%w/v). The higher rate of drug release from the Carbopol gel base (88.056 ± 0.04) can be attributed to the pores within the gel, which facilitate the free diffusion of the drug into the surrounding medium. Additionally, the solubility of lipophilic drugs in aqueous environments is within minimal limits to ensure their availability for release. In contrast, drug release from 1% w/v Carbopol was recorded at 82.78 ± 0.15 after the same duration. A significant decrease in drug release (P˂0.05) was detected when the Carbopol concentration was increased from 0.5% to 1% w/v. These findings suggest that polymer selection and concentration play crucial roles in modulating IVR release.

The developed IVR gels showed release rates that most closely matched the simplified Higuchi equation, as evidenced by the highest correlation coefficient (r²). This kinetic behavior suggests that drug penetration occurs primarily through diffusion, which follows a diffusion-controlled mechanism. The drug diffuses through the polymer network channels within the gel while also exhibiting slow and sustained penetration through the skin membrane.

The novelty of this work lies in presenting an ivermectin (IVR) hydrogel formulation as an advanced topical delivery system. Unlike conventional therapies, hydrogel enhances skin penetration, and incorporates anti-inflammatory and antioxidant properties, offering a potential well-tolerated scabicide, this unique formula helps to avoid the side effects on subcutaneous ivermectin injection with a more effective results in clinical, histopathological and serological levels. Comparable research, including crotamiton-loaded microemulsion hydrogels, has shown improved dermal penetration and safety, supporting the potential of hydrogel-based systems for scabies management^107^. In addition, a previous study done by Dahlizar et al.^31^ who reported that Pal-GH gel spray formulation could be a promising topical IVR formulation for scabies, owing to its efficacy, high skin permeation, and the practical advantage of large-area application without prolonged contact. However, this study lacked direct acaricidal testing or clinical efficacy validation. Our multi-parameter evaluation of disease progression in this model provides comprehensive evidence positioning the hydrogel as a clinically promising formula.

## Study limitations

The present study revealed some limitations, including the need for investigating the ovicidal activity of this formulation, besides comparative testing against commercially available topical permethrin in vivo bioassays to determine their relative efficacy, compliance, and cost-effectiveness. Several challenges may influence its real-world clinical translation. One of the primary considerations is manufacturing scalability and cost-effectiveness, particularly in comparison to conventional formulations such as permethrin creams, which are widely available and relatively inexpensive. In addition, the 21-day treatment regimen may not effectively measure long-term relapse rates or delayed adverse consequences; thus, long-term evaluation would be valuable. Scratching behavior and pruritus can be highly variable and are not feasible to quantify reliably with the resources available. Future studies could incorporate validated itch scales or electronic activity monitors to complement the clinical and parasitological data.

## Conclusions

This study developed and evaluated a novel topical hydrogel formulation of ivermectin (IVR) for the treatment of scabies. An optimized 3% w/v HPMC-based hydrogel demonstrated an effective anti scabies activity consistent with Higuchi diffusion kinetics. In vitro, the formulation exhibited significant, concentration-dependent acaricidal activity against *S. scabiei* mites, achieving 100% mortality within 10 min. In an infected rabbit model, a 21-day topical treatment regimen led to significant clinical (the general condition of rabbits and complete healing of lesions and disappearance of crusts) and histopathological improvement, including resolution of skin lesions and reductions in hyperkeratosis and inflammation. Regarding safety, IVR hydrogel could be considered a well-tolerated application, reducing MDA more effectively, with no adverse behavioral alterations beyond infection-related changes. While subcutaneous IVR, conversely, elevated MDA, consistent with reports of transient oxidative effects post-systemic dosing. These findings focus on the potency of this developed hydrogel as a very effective and safer alternative to conventional systemic treatment. These trends may imply a favourable topical safety profile in this model, but long-term toxicity, skin irritation, and human pharmacokinetics remain unassessed, precluding broad safety claims. Whereas the present IVR topical formulation shows promising results but may not replace oral IVR in advanced cases where systemic therapy is warranted, emphasizing the need for combination therapy of both topical and systemic treatment to achieve maximum results. In addition, further clinical trials are encouraged to assess the long-term safety, the mechanism of action, and evaluate its efficacy in human scabies, especially in severe cases, besides the evaluation of permeation enhancer optimization before human use.

## Supplementary Information

Below is the link to the electronic supplementary material.

Supplementary Material 1

## Data Availability

All data are available within the manuscript without restriction.:

## Abbreviations

IVR: Ivermectin
HPMC: Hydroxypropylmethyl cellulose
Na-alginate: Sodium alginate
SEM: Scanning electron microscopy
LC_50_: Lethal concentration responsible for death of 50% of mites
LT_50_: Median lethal times
TNF-α: Tumor necrosis factor alpha
IL-6: Interleukin-6
MDA: Malondialdehyde
IgE: Immunoglobulin E
IgG: Immunoglobulin G
