# Supplementary figures and images for: Integrated in vitro and in vivo evaluation of ivermectin hydrogel formulation for management of scabies with pharmacological assessment
Source: Sci Rep. 2026 Jun 4;16:17327. doi: 10.1038/s41598-026-53626-w (PMC13237114; doi:10.1038/s41598-026-53626-w)

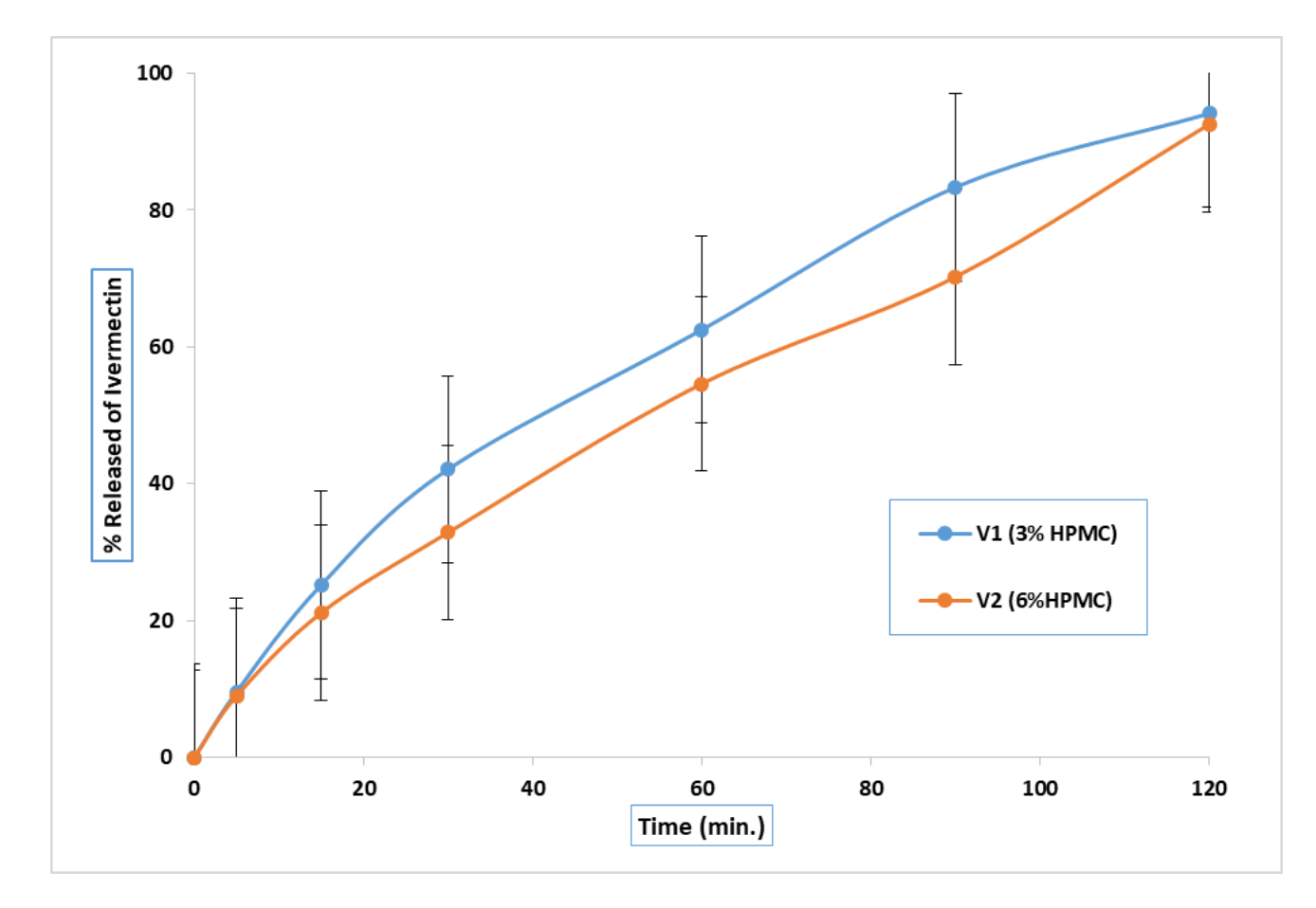

Supplement: Supplementary file 1 — Supplementary Material 1 [file 41598_2026_53626_MOESM1_ESM.zip › figures upload/fig 1.tif]

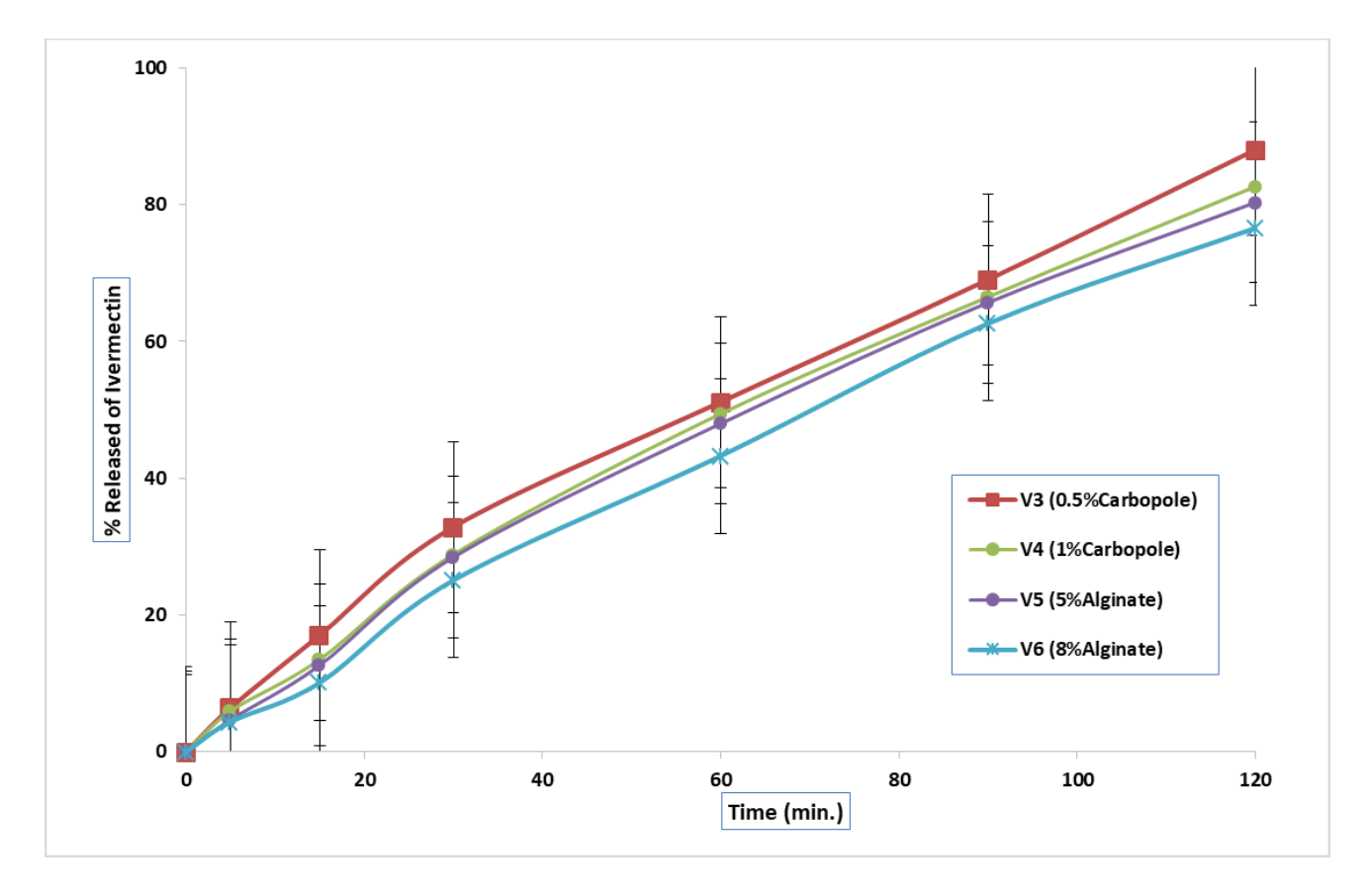

Supplement: Supplementary file 1 — Supplementary Material 1 [file 41598_2026_53626_MOESM1_ESM.zip › figures upload/fig 2.tif]

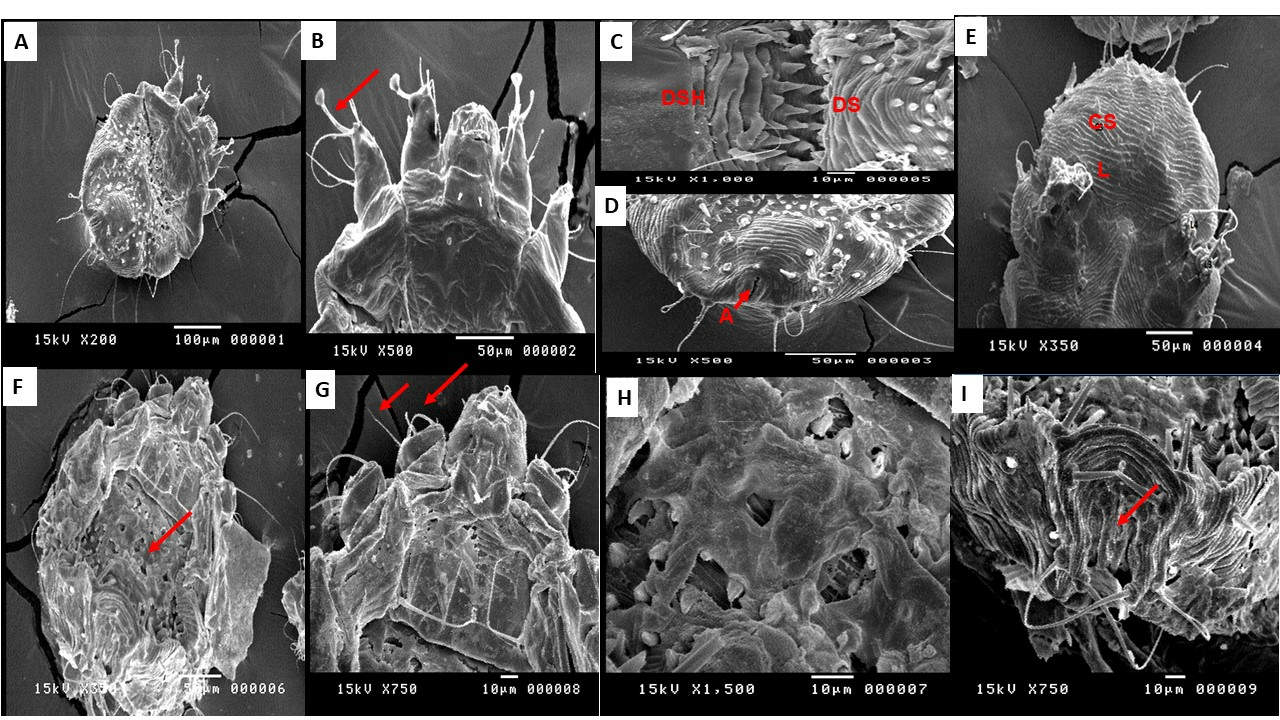

Supplement: Supplementary file 1 — Supplementary Material 1 [file 41598_2026_53626_MOESM1_ESM.zip › figures upload/FIG 4.tif]

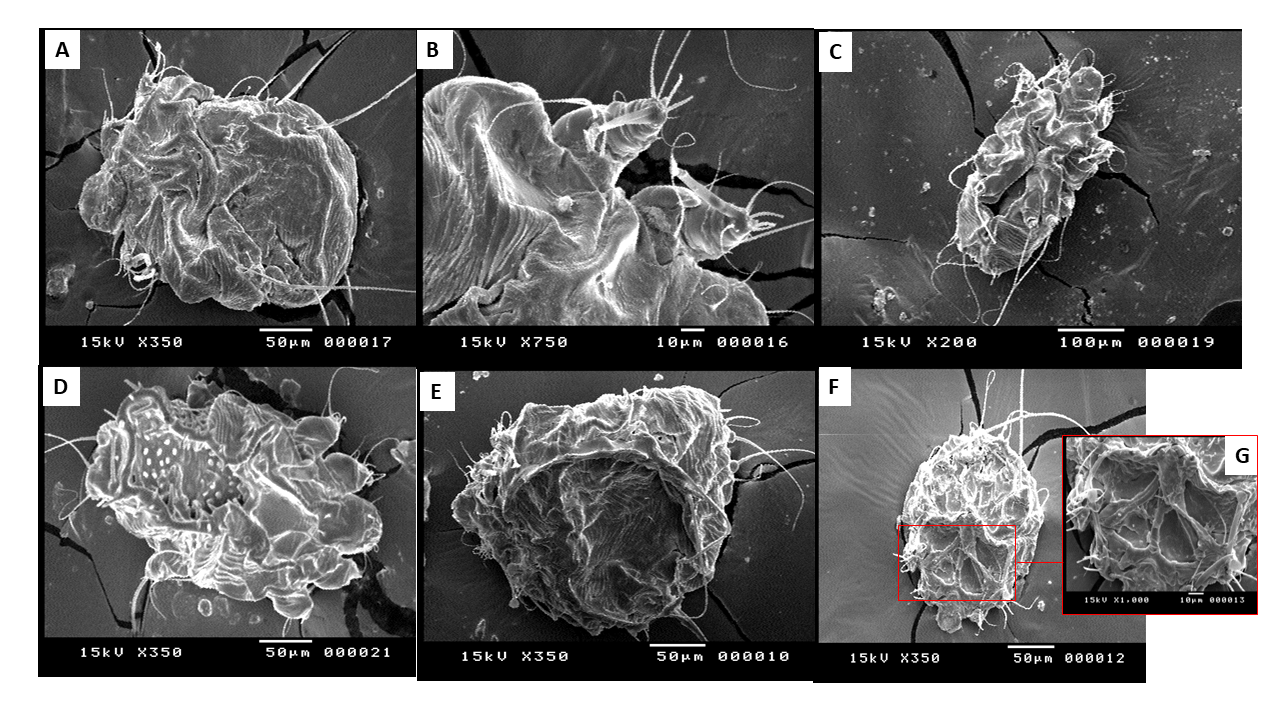

Supplement: Supplementary file 1 — Supplementary Material 1 [file 41598_2026_53626_MOESM1_ESM.zip › figures upload/fig 5.TIF]

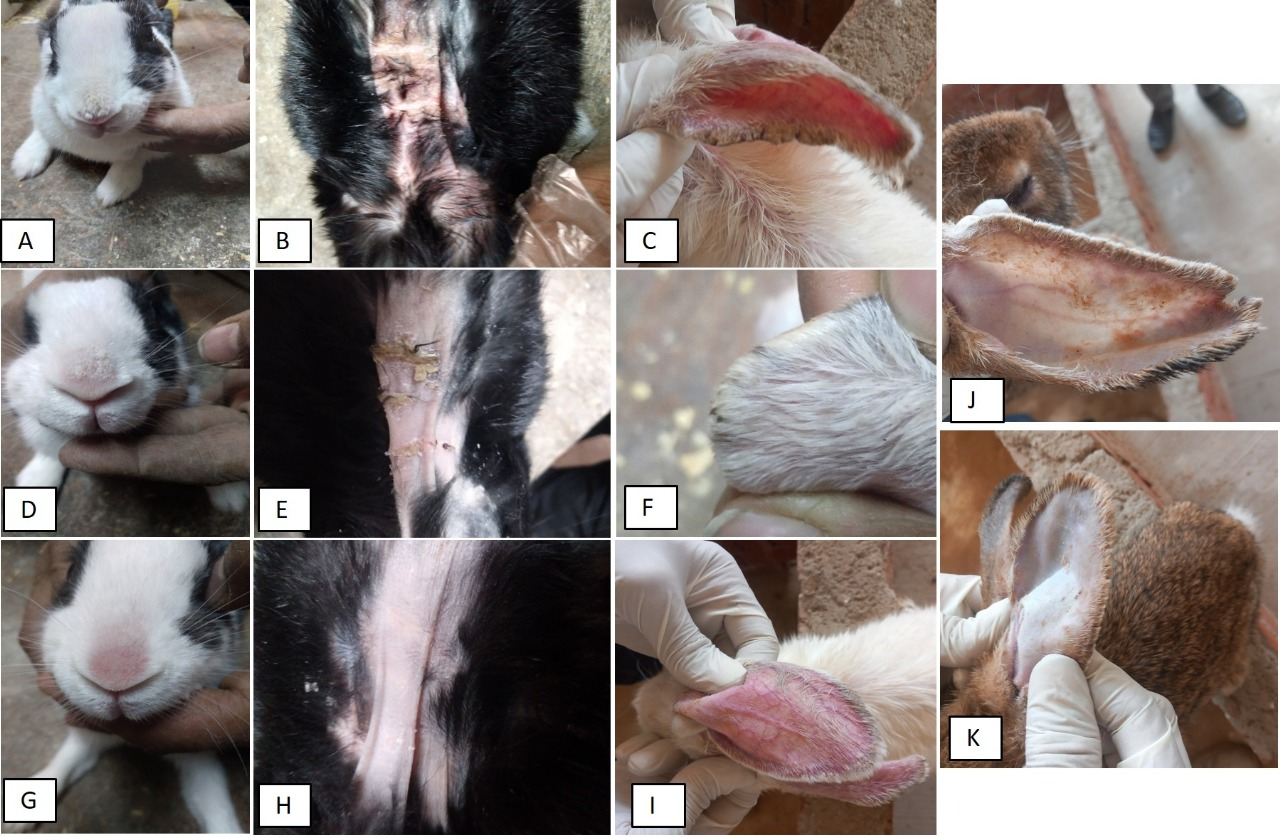

Supplement: Supplementary file 1 — Supplementary Material 1 [file 41598_2026_53626_MOESM1_ESM.zip › figures upload/FIG 6.tif]

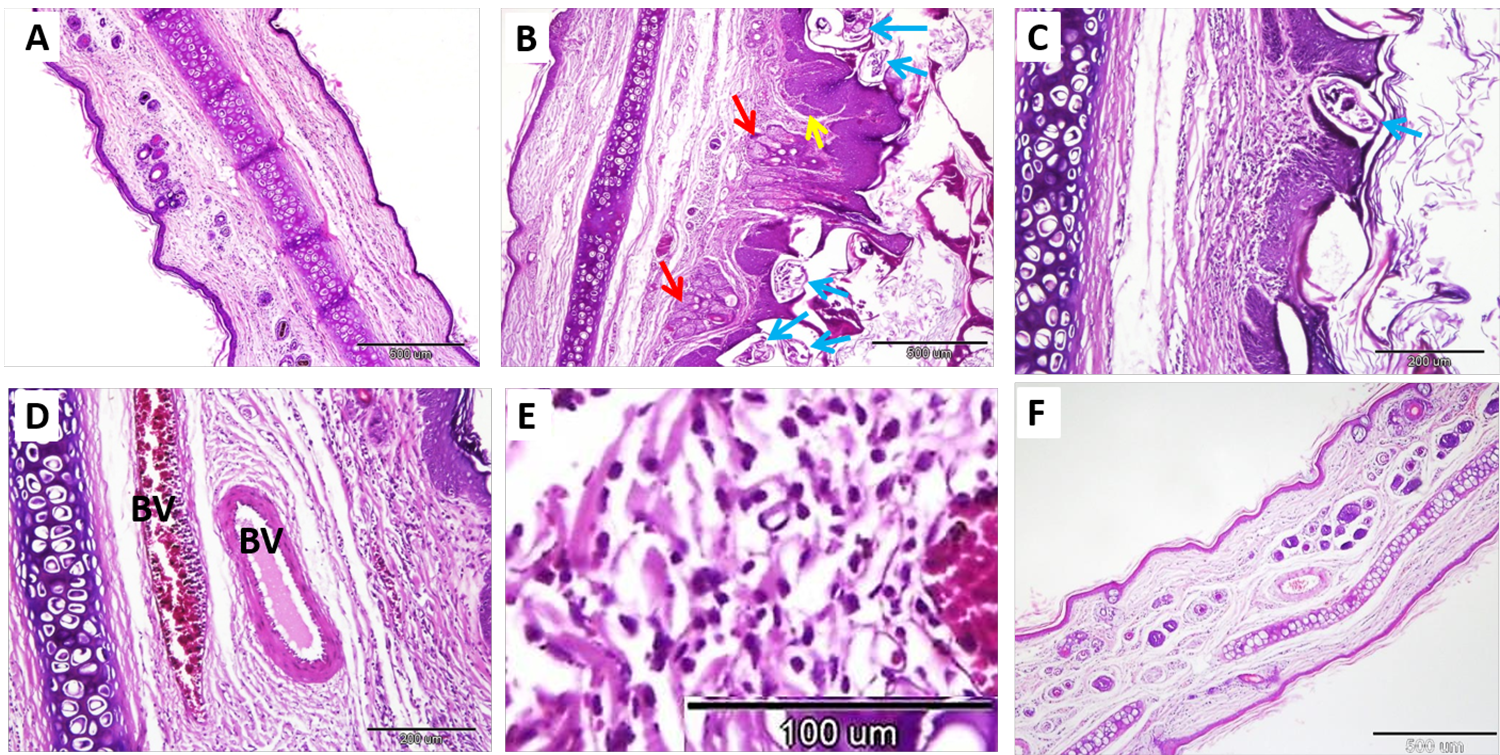

Supplement: Supplementary file 1 — Supplementary Material 1 [file 41598_2026_53626_MOESM1_ESM.zip › figures upload/fig 7.tif]

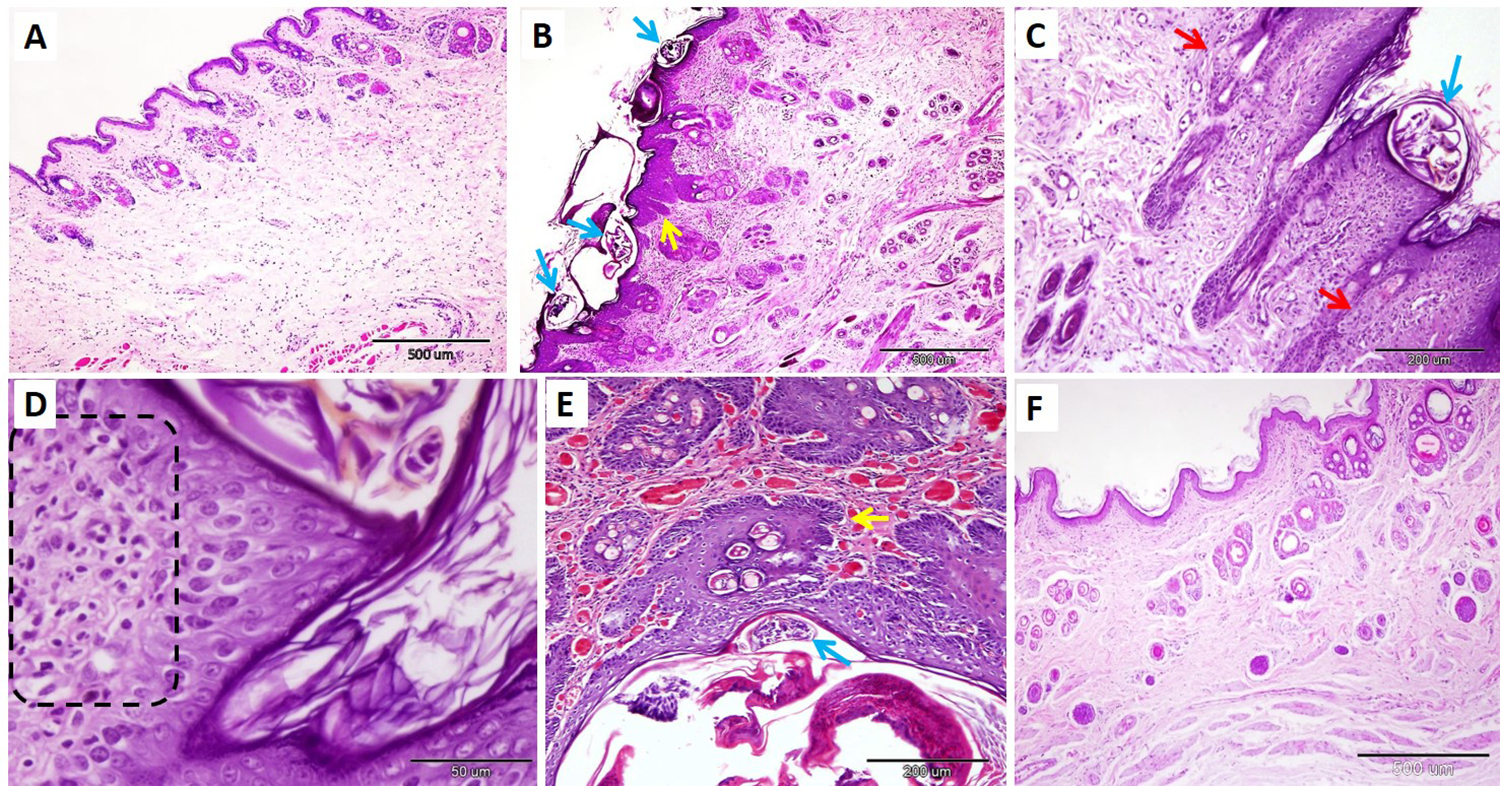

Supplement: Supplementary file 1 — Supplementary Material 1 [file 41598_2026_53626_MOESM1_ESM.zip › figures upload/fig 8.tif]

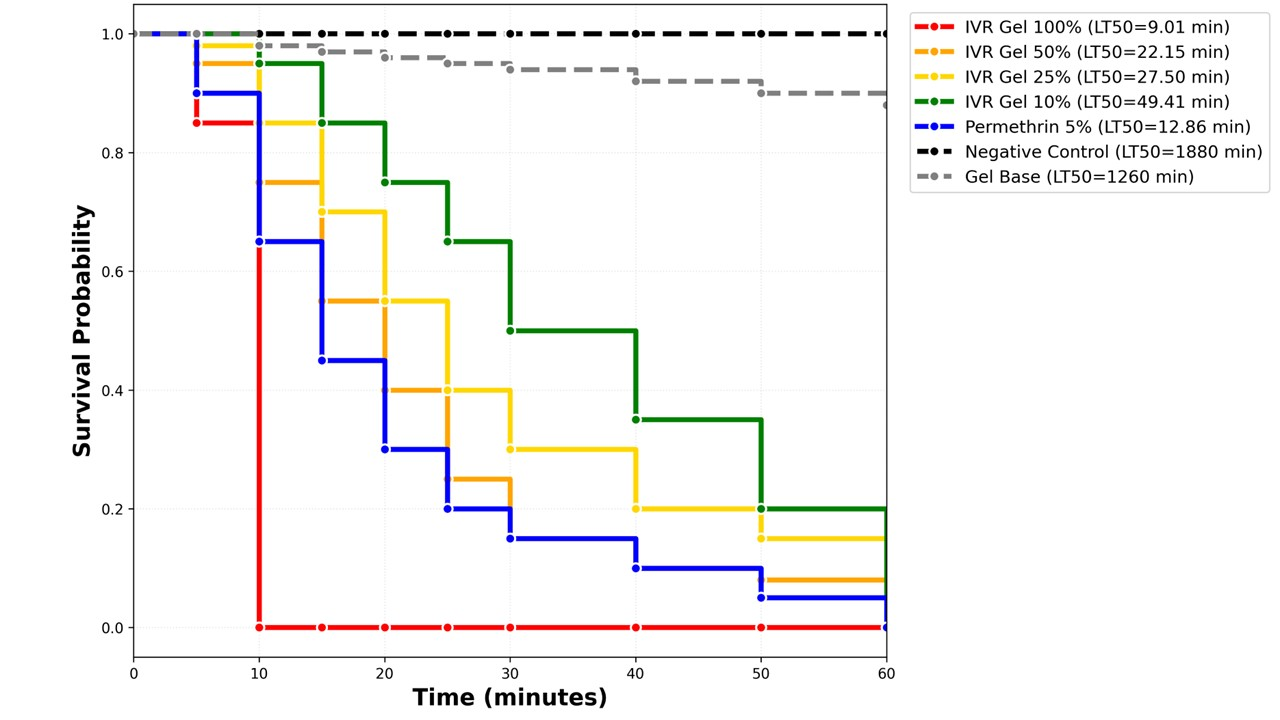

Supplement: Supplementary file 1 — Supplementary Material 1 [file 41598_2026_53626_MOESM1_ESM.zip › figures upload/Fig 3.tif]
